# Phenolic Metabolites from a Deep-Sea-Derived Fungus *Aspergillus puniceus* A2 and Their Nrf2-Dependent Anti-Inflammatory Effects

**DOI:** 10.3390/md20090575

**Published:** 2022-09-13

**Authors:** Jianlin He, Xin Wu, Shuhuan Huang, Juan Wang, Siwen Niu, Meixiang Chen, Gaiyun Zhang, Songyan Cai, Jingna Wu, Bihong Hong

**Affiliations:** 1 Third Institute of Oceanography, Ministry of Natural Resources, Xiamen 361005, China; 2 Technical Innovation Center for Exploitation of Marine Biological Resources, Ministry of Natural Resources, Xiamen 361005, China; 3 Fujian Provincial Key Laboratory of Island Conservation and Development, Island Research Center, Ministry of Natural Resources, Pingtan 350400, China; 4 Department of Pharmacy, Xiamen Medical College, Xiamen 361023, China

**Keywords:** Nrf2 activator, Raw264.7, phenolic metabolites, asperpropanols, COX-2, iNOS

## Abstract

Four undescribed phenolic compounds, namely asperpropanols A–D (**1**–**4**), along with two known congeners **5** and **6**, were isolated from *Aspergillus puniceus* A2, a deep-sea-derived fungus. The gross structures of the compounds were established by detailed analyses of the HRESIMS and NMR data, and their absolute configurations were resolved by modified Mosher’s method and calculations of ECD data. Compounds **1**–**6** were found to have excellent anti-inflammatory effect on lipopolysaccharide (LPS)-induced RAW264.7 cells at 20 μM, evidenced by the reduced nitric oxide (NO), tumor necrosis factor α, and interleukin 6 production. Among them, **5** and **6** showed inhibitory effects on NO production comparable with the positive control (BAY11-7083 at 10 μM). Additionally, the LPS-induced mRNA expressions of inducible nitric oxide synthase and cyclooxygenase-2 were also decreased. Interestingly, mRNA expression of nuclear factor erythroid 2–related factor 2 (Nrf2) was downregulated by LPS and recovered by **1**–**6**, suggesting a vital role of Nrf2 in their effect. We further found that pharmacological inhibition of Nrf2 by ML385 largely abrogated the effects of **1**–**6** on RAW264.7 cells. Therefore, **1**–**6** may share a common anti-inflammatory mechanism via Nrf2 upregulation and activation.

## 1. Introduction

*Aspergillus* fungi are distributed in nearly every marine habitat explored, and their metabolites encompass a great diversity in both chemistry and bioactivity [1]. The metabolites of *Aspergillus* include polyketides, alkaloids, terpenoids and other structural types, which have biological activities such as antibacterial, antitumor, and metabolic regulation [2]. For example, the well-known cholesterol-lowering agent lovastatin was found in a fermentation broth of *Aspergillus terreus* [3]. As for the marine-derived *Aspergillus*, the firstly reported novel compounds were fumiquinazolines A-C, discovered by Numata et al. in 1992 [4]. Since then, the number of novel compounds from marine-derived *Aspergillus* has been increasing each year, and so far, more than 500 novel compounds have been discovered from marine-derived *Aspergillus* [5].

**Figure 1:**
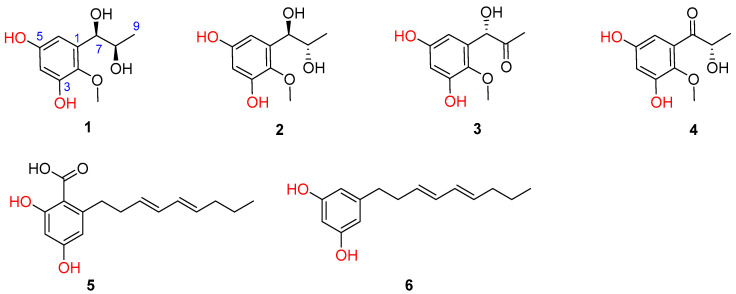
Chemical structures of **1**–**6** isolated from *A. puniceus* A2.

Previously, eight novel diketopiperazine-type alkaloids were isolated from a deep-sea-derived fungus *Aspergillus puniceus* SCSIO z021, and they were found to be LXRα agonists with the EC50 values of 1.7−50 μM [6]. Later, the same team found nine new mycotoxins (xanthone-type and anthraquinone-type) and fourteen new isoquinoline alkaloids as inhibitors of protein tyrosine phosphatases in the extract of the strain [7,8]. *Aspergillus puniceus* A2 was newly isolated from the deep-sea sediment, and its secondary metabolites were investigated in order to find novel and bioactive compounds. Under our continuous efforts, four novel phenolic metabolites and two known congeners were isolated from the fermentation broth of *A. puniceus* A2 (Figure 1). 

Inflammation is a basic response to pathogens, toxic compounds and damaged cells. It is triggered by the immune system, and is an essential part of self-healing [9]. However, chronic inflammation caused by lifestyle, genetic, environmental and other factors can be detrimental. Therefore, inflammation is associated with a wide range of diseases such as arteriosclerosis, type 2 diabetes, fatty liver disease, asthma, obesity, Alzheimer’s and Parkinson’s diseases, inflammatory bowel disease and cancers [10]. Herein, the anti-inflammatory effect of the isolated compounds **1–6** from *A. puniceus* A2 were investigated in a lipopolysaccharide (LPS)-stimulated Raw264.7 cell model. Additionally, the mechanism underlying their effect was explored and a common regulator was revealed. Our discoveries may provide a novel insight into marine-derived compounds with therapeutic implications for inflammatory-related diseases.

## 2. Results and Discussion

### 2.1. Chemistry 

**Figure 2:**
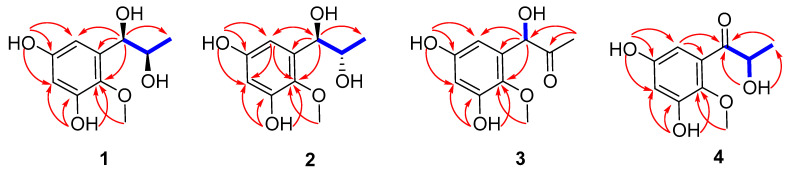
Selected ^1^H-^1^H COSY (blue bold) and HMBC (red arrow) correlations of **1**–**4**.

**Figure 3:**
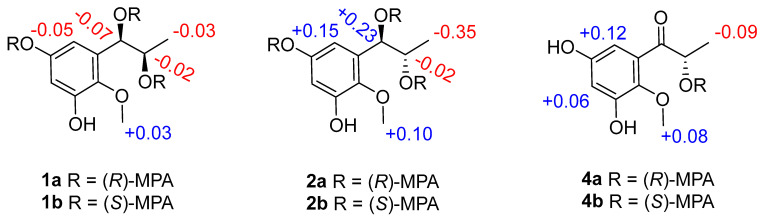
Δ*δ*_H_ values (*δ*_R_ − *δ*_S_) of the MPA esters of **1**, **2** and **4** in CDCl_3_.

Asperpropanol A (**1**) was isolated as a brownish red powder, and its molecular formula was determined to be C_10_H_14_O_5_ on the basis of the sodium adduct ion peak at *m/z* 237.0727 [M + Na]^+^ in the HRESIMS spectrum, requiring four degrees of unsaturation. The IR spectrum exhibited absorption bands for OH (3425 cm^−1^) and benzene (1604 cm^−1^) functional groups. The ^1^H NMR spectrum of **1** displayed two aromatic protons at *δ*_H_ 6.20, two oxymethines (*δ*_H_ 3.60, 4.48), two methyls (*δ*_H_ 0.87, 3.62) and four exchangeable protons (*δ*_H_ 4.49, 4.94, 8.86 and 9.07) (Table 1), while its ^13^C NMR spectrum exhibited 10 resonance signals involving 6 aromatic carbons (*δ*_C_ 103.0, 104.7, 137.2, 138.3, 150.4 and 153.6) for a phenyl moiety, two oxygenated methines (*δ*_C_ 71.2 and 72.3), one methyl (*δ*_C_ 19.8), and a methoxy group (*δ*_C_ 60.5) (Table 2). In the ^1^H-^1^H COSY spectrum, the cross-peaks from H-8 (*δ*_H_ 3.60) to H_3_-9 (*δ*_H_ 0.87), H-7 (*δ*_H_ 4.48) and 8-OH (*δ*_H_ 4.49), and from H-7 to 7-OH (*δ*_H_ 4.94) deduced the presence of a 1,2-dihydroxypropane unit (Figure 2). In addition, the HMBC correlations from 3-OH (*δ*_H_ 9.07) to C-2 (*δ*_C_ 138.3), C-3 (*δ*_C_ 150.4) and C-4 (*δ*_C_ 103.0), from 5-OH (*δ*_H_ 8.86) to C-4, C-5 (*δ*_C_ 153.6) and C-6 (*δ*_C_ 104.7), from H-7 to C-1 (*δ*_C_ 137.2)/C-2/C-6 and from OCH_3_ (*δ*_H_ 3.62) to C-2 deduced two hydroxy groups, a methoxy group and a 1,2-dihydroxypropane unit, which were substituted at C-3, C-5, C-2 and C-1, respectively, of the benzene ring. Therefore, the gross structure of **1** was assigned to be 1-(3′,5′-dihydroxy-2′-methoxyphenyl)propane-1,2-diol. In order to establish the absolute configuration of 1,2-diol unit (C-7 and C-8) in the flexible side chain, the modified Mosher’s method was used [11]. Compound **1** was reacted separately with (*R*)- and (*S*)- MPA to obtain (*R*)-MPA ester (**1a**) and (*S*)-MPA ester (**1b**), respectively. The Δ*δ*_H_ values of H-7 (–0.07) and H-8 (–0.02) between **1a** and **1b** (Δ*δ*_H_ = *δ*_R_ − *δ*_S_) (Figure 3) secured the *R* configurations at C-7 and C-8 according to the acyclic *syn*-1,2-diol Type B [11]. Thus, the structure of **1** was elucidated as a new phenylpropanoid with four hydroxy groups at C-3, C-5, C-7 and C-8, as well as a methoxy group at C-2. HRESIMS, ^1^H, ^13^C, HSQC, COSY, HMBC and IR spectra of **1** were provided in Supplementary Figures S1–S6 and S9, and ^1^H NMR spectra of (R)- and (S)- MPA esters of **1**(**1a)** and **1(1b)** were provided in Supplementary Figures S7 and S8.

The molecular formula of compound **2** was deciphered to be the same as that of **1** on the basis of the HRESIMS spectrum and ^13^C NMR data. The IR spectrum exhibited absorption bands for OH (3414 cm^−1^) and benzene (1610 and 1476 cm^−1^) functional groups. The ^1^H and ^13^C NMR data of **2** were comparable to those of **1**, revealing that they were structurally similar congeners. However, **2** displayed the deshielded chemical shifts at H-7 (*δ*_H_ 4.65), H-8 (*δ*_H_ 3.68) and H_3_-9 (*δ*_H_ 0.95) and shielded shifts at C-7 (*δ*_C_ 71.5), C-8 (*δ*_C_ 69.9) and C-9 (*δ*_C_ 17.9) compared to the corresponding resonances of **1**. Analyses of the 2D NMR data determined the identical planar structure between **2** and **1**, as evidenced by the COSY correlations of 7-OH (*δ*_H_ 4.79)/H-7/H-8/8-OH (*δ*_H_ 4.36) and H-8/H_3_-9, in association with the HMBC cross-peaks from 3-OH (*δ*_H_ 8.97) to C-2 (*δ*_C_ 138.2)/C-3 (*δ*_C_ 150.2)/C-4 (*δ*_C_ 102.8), from 5-OH (*δ*_H_ 8.79) to C-4/C-5 (*δ*_C_ 153.4)/C-6 (*δ*_C_ 104.7), from H-6 (*δ*_H_ 6.27) to C-2/C-4/C-7 and from OCH_3_ (*δ*_H_ 3.61) to C-2 (Figure 2). Finally, the absolute configurations of 7,8-diol moiety were also resolved on the basis of the modified Mosher’s method. Analyses of the Δ*δ*_H_ signs between the MPA esters of **2a** and **2b** at H_3_-9 (−0.35), H-8 (−0.09), H-7 (+0.23), H-6 (+0.15) and 2-OCH_3_ (+0.10) assigned the 7*R* and 8*S* configurations (Figure 3). Therefore, the structure of **2** was determined to be a C-8 epimer of **1** and given the trivial name asperpropanol B. HRESIMS, ^1^H, ^13^C, HSQC, COSY, HMBC and IR spectra of **2** were provided in Supplementary Figures S10–S15 and S18, and ^1^H NMR spectra of (R)- and (S)- MPA esters of **2**(**2a**) and **2**(**2b**) were provided in Supplementary Figures S16 and S17.

**Figure 4:**
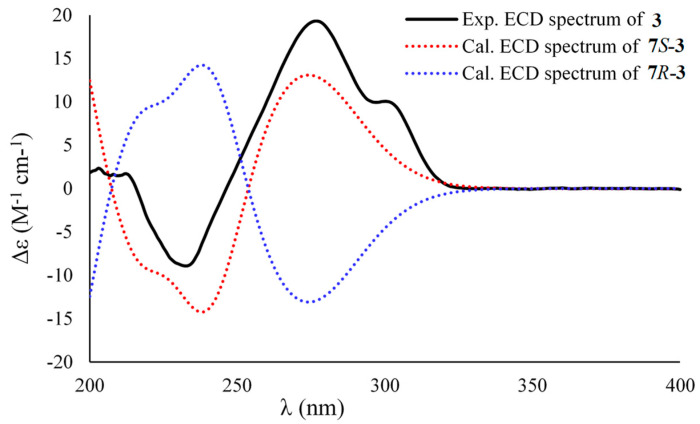
Experimental ECD spectrum of **3** in methanol and the calculated ECD data of 7*S*-**3** and 7*R*-**3**.

Asperpropanol C (**3**) had a molecular formula of C_10_H_12_O_5_ as established by the positive HRESIMS and ^13^C NMR spectra, indicating five indices of hydrogen deficiency. The IR spectrum exhibited absorption bands for OH (3423 cm^−1^), C=O (1715 cm^−1^) and benzene (1612 and 1510 cm^−1^) functional groups. The NMR spectroscopic data of **3** closely resembled those of **1** and **2**, except for the presence of a ketone carbonyl carbon (*δ*_C_ 208.3) and the absence of an oxygenated methine group in **3**. The observations in association with the presence of a singlet methyl (*δ*_H_ 2.03) in **3** instead of the doublet methyl in **1** and **2** deduced that the keto group was located at C-8. This assumption was corroborated on the basis of the HMBC interactions from H_3_-9 (*δ*_H_ 2.03) to C-7 (*δ*_C_ 74.7) and C-8 (*δ*_C_ 208.3) and from H-7 (*δ*_H_ 5.10) to C-1 (*δ*_C_ 134.2), C-2 (*δ*_C_ 138.5), C-6 (*δ*_C_ 105.2), C-8 and C-9 (*δ*_C_ 26.0) (Figure 2). To resolve the absolute configuration of the sole chiral center of C-7, the theoretically calculated ECD spectra of 7*S*-**3** and 7*R*-**3** were carried out using the Gaussian 09 program [12]. The conformational search was performed by the BARISTA 7.0 software with the MMFF94S force field within an energy window of 5.0 kcal/mol. All the conformers were optimized at the 6-31+G(d,p) level in the gas phase. The optimized conformers with a Boltzmann population over 1% were further calculated at the B3LYP/6-311+G(2d, p) level with the CPCM model in methanol. As shown in Figure 4, the calculated ECD data of 7*S*-**3** matched well with the experimental ECD curves of **3** in methanol, indicating the 7*S* configuration, which was further supported by the opposite optical rotation data between **3** ([*α*${]}_{D}^{\mathrm{23}}$ +73, MeOH) and (*R*)-(–)-1-(3,5-dihydroxyphenyl)-1-hydroxypropane-2-one ([*α*${]}_{D}^{23}$ −230, MeOH) [13]. HRESIMS, ^1^H, ^13^C, HSQC, COSY, HMBC and IR spectra of **3** were provided in Supplementary Figures S19–S25.

Asperpropanol D (**4**) had the same molecular formula (C_10_H_12_O_5_) as that of **3**, as determined by the HRESIMS and NMR data. The IR spectrum exhibited absorption bands for OH (3427 cm^−1^), C=O (1684 cm^−1^) and benzene (1610 and 1467 cm^−1^) functional groups. The ^1^H and ^13^C NMR data of **4** were nearly identical to those of **3** except that the singlet methyl of **3** was replaced by a doublet in **4**. The obvious difference indicated the keto group to be positioned at C-7 in **4** instead of C-8 in **3**, as demonstrated by the COSY cross-peaks from H-8 (*δ*_H_ 4.76) to H_3_-9 (*δ*_H_ 1.17) and 8-OH (*δ*_H_ 5.08), together with the HMBC correlations from H_3_-9 to carbonyl carbon C-7 (*δ*_C_ 205.6) and C-8 (*δ*_C_ 71.8), from 8-OH to C-7/C-8/C-9 (*δ*_C_ 20.4), and from H-6 (*δ*_H_ 6.25) to C-2 (*δ*_C_ 139.0), C-5 (*δ*_C_ 153.9) and C-7 (Figure 2). The absolute configuration of the stereogenic center of C-8 was resolved on the basis of the modified Mosher’s method [14]. Compound **4** was esterified separately with (*R*)- and (*S*)-MPA to yield (*R*)-MPA derivative **4a** and (*S*)-MPA derivative **4b**, respectively. Analyses of the ^1^H NMR chemical shift differences between **4a** and **4b** (Δ*δ* = *δ*_R_ − *δ*_S_) confirmed the 8*S* configuration (Figure 3). HRESIMS, ^1^H, ^13^C, HSQC, COSY, HMBC and IR spectra of **4** were provided in Supplementary Figures S26–S31 and S34, and ^1^H NMR spectra of (R)- and (S)- MPA esters of **4**(**4a**) and **4**(**4b**) were provided in Supplementary Figures S32 and S33.

Furthermore, two additional known phenolic metabolites were assigned to be 2,4-dihydroxy-6-((3*E*,5*E*)-nona-3,5-dien-1-yl)-benzoic acid (**5**) [15,16] and 5-[(3*E*,5*E*)-3,5-nonadienyl]-1,3-benzenediol (**6**) [16], respectively, on the basis of the careful comparisons of their NMR data and specific rotations with those reported in the literature.

### 2.2. Biology

**Figure 5:**
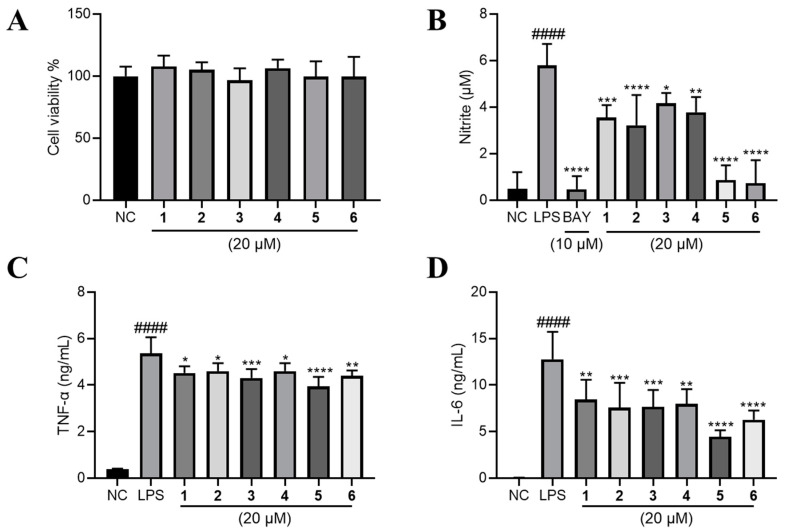
The effects of compounds **1**–**6** on LPS-induced inflammation on RAW264.7 cells. (**A**) The effect of **1**–**6** on the cell viability of RAW264.7 cells. LPS (1 μg/mL) was added to all groups except normal control (NC, 0.2% DMSO, *v*/*v*). The production of (**B**) NO, (**C**) TNF-α and (**D**) IL-6 were measured. Mean ± SD (*n* = 6); #### *p* < 0.0001 LPS vs. NC; * *p* < 0.05, ** *p* < 0.01, *** *p* < 0.001, **** *p* < 0.0001 vs. LPS.

Raw264.7 cells were employed to evaluate the anti-inflammatory effect of compounds **1–6.** We firstly investigated their cytotoxic effect, and found that none of them have significant inhibitory effect on the cell viability of Raw264.7 cells at the concentration of 20 μM (Figure 5A). Next, we found that LPS-induced nitric oxide (NO) production was significantly lowered by **1**–**6** at 20 μM. Among them, **5** and **6** exhibited comparable effect to BAY11-7083 (BAY, the positive control) of 10 μM (Figure 5B). Major inflammatory cytokines tumor necrosis factor-α (TNF-α) and interleukin 6 (IL-6) were also robustly elevated by LPS and significantly lowered by the treatment of compounds **1**–**6** (Figure 5C,D).

**Figure 6:**
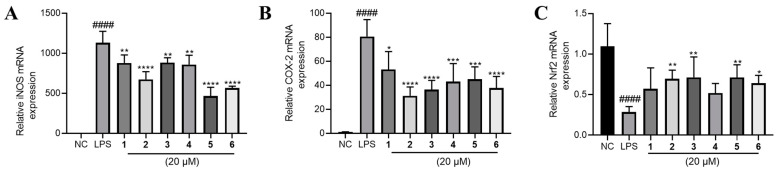
The effects of compounds **1**–**6** on mRNA expression of (**A**) iNOS, (**B**) COX-2 and (**C**) Nrf2 in LPS-stimulated RAW264.7 cells. LPS (1 μg/mL) was added to all groups except normal control (NC, 0.2% DMSO, *v*/*v*). Mean ± SD (*n* = 6); #### *p* < 0.0001 LPS vs. NC; * *p* < 0.05, ** *p* < 0.01, *** *p* < 0.001, **** *p* < 0.0001 vs. LPS.

The above results indicated that all the tested phenolic metabolites had anti-inflammatory effect. To explore the underlying mechanism, we determined the mRNA expression of several key inflammation-related genes. In consequence, it was found that inducible nitric oxide synthase (iNOS) and cyclooxygenase-2 (COX-2) expression were sharply elevated by LPS treatment and significantly lowered by **1**–**6** (Figure 6A,B). 

NO is a free radical with an unpaired electron, and iNOS produces log-fold higher amounts of NO as an immune defense mechanism [17]. COX-2 was reported to elevate IL-6 in a pristane-treated murine model of inflammation [18]. Inhibition of COX-2 reduced IL-6 expression in both cell and animal models [19,20]. The literatures suggest a synergistic pro-inflammatory effect of COX-2 and TNF-α [21,22]. Therefore, the result of the alteration of the expression of pro-inflammatory genes iNOS and COX-2 were consistent with the regulation of NO, TNF-α and IL-6 production (Figure 5). 

**Figure 7:**
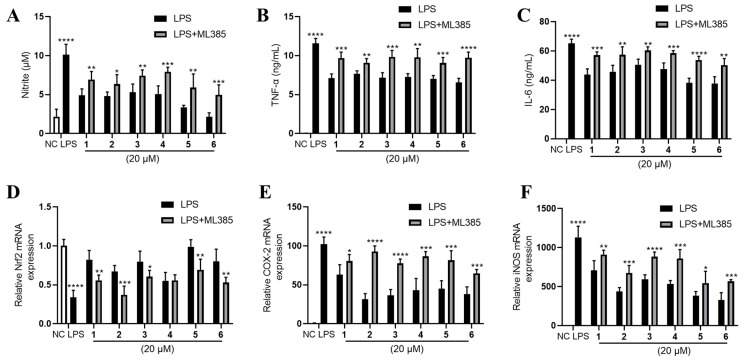
The Nrf2-dependent anti-inflammation effect of **1**–**6** on LPS-stimulated RAW264.7. (**A**) NO production, (**B**) TNF-α and (**C**) IL-6 were measured. mRNA expression of (**D**) Nrf2, (**E**) COX-2 and (**F**) iNOS were measured. LPS (1 μg/mL) was added to all groups except normal control (NC, 0.25% DMSO, *v*/*v*). ML385 was added at 5 μM. Mean ± SD (*n* = 6); * *p* < 0.05, ** *p* < 0.01, *** *p* < 0.001, **** *p* < 0.0001 LPS vs. NC, LPS + ML385 vs. LPS.

Furthermore, we found that Nrf2 was inhibited by LPS and partly rescued by the phenolic metabolites’ treatment (Figure 6C). Since Nrf2 is a master regulator that protects against oxidative damage and inflammatory responses [23], we inferred from the above result that the effect of **1**–**6** may be Nrf2-dependent. Hence, we used a specific Nrf2 inhibitor ML385 to observe the corresponding alteration of the inflammatory phenotypes under the treatment of **1**–**6**. Intriguingly, the inhibitory effects of **1**–**6** on NO, TNF-α and IL-6 production were largely abolished by the antagonism of Nrf2 by ML385 (Figure 7A–C). The upregulation of Nrf2 by the compounds also partly diminished except for compound **4** (Figure 7D). Furthermore, the downregulation of COX-2 and iNOS by **1**–**6** treatment was abrogated to a large extent (Figure 7E,F). 

Nrf2 is a transcript factor that binds to antioxidant response element (ARE) to activate the downstream genes involved in antioxidative and anti-inflammatory defenses [24]. ML385 interacts with Nrf2, and most likely blocks the binding of NRF2 to AREs [25]. Since ML385 largely abolished the anti-inflammatory effect of **1**–**6**, we inferred that **1**–**6** may activate Nrf2 to exert their anti-inflammatory effect in LPS-stimulated RAW264.7 cells. It should be noted that **1**–**6** upregulated the expression of Nrf2 (Figure 5C), which may also contribute to their anti-inflammatory effect.

## 3. Materials and Methods

### 3.1. General Experimental Procedures

The UV data were measured on a UV-1800 spectrophotometer (Shimadzu, Kyoto, Japan). Optical rotation data were acquired on an MCP 500 automatic polarimeter (Anton Paar, Graz, Austria). The ECD spectra were recorded on a Chirascan Circular Dichroism Spectrometer (Applied Photophysics, Leatherhead, UK). The IR spectra were recorded on a Tensor27 FT-IR spectrophotometer (Bruker Optics, Ettlingen, Germany). The ^1^H, ^13^C, HSQC, COSY and HMBC spectra were recorded on an AV-600 MHz spectrometer (Bruker, Karlsruhe, Germany), and the NMR chemical shifts (*δ*) were referenced to the solvent peaks of DMSO-*d*_6_ at 2.50 and 39.5 ppm for proton and carbon, respectively. The HRESIMS spectra were acquired on the basis of the Xevo G2 Q-TOF mass spectrometer (Waters, Manchester, UK). Semipreparative HPLC was conducted on an Alltech LS class pumpequipped with UV/Vis detector (Alltech, Deerfield, IL, USA), and a packed column (ODS-A, 250 × 10 mm, 5 μm, Cosmosil, Kyoto, Japan) was used for the isolation and purification. Column chromatography (CC) was performed on the basis of Sephadex LH-20 (Pharmacia, Uppsala, Sweden), silica gel and ODS (Osaka Soda, Tokyo, Japan). Precoated silica gel plates GF-254 (Jiangyou Silicon Development, Yantai, China) were used for TLC analysis. All solvents used for CC were analytical grade.

### 3.2. Fungal Material and Fermentation

The producing fungus, which was obtained from the deep-sea sediment at the depth of 4841 m sampling from the Pacific Ocean, was identified to be *Aspergillus puniceus* on the basis of its ITS gene sequence, which exhibited 99.83% homology to those of the *Aspergillus puniceus* SRRC 2155 (accession no. AY373863). Thus, the fungus was named *Aspergillus puniceus* A2, and the ITS sequence information was deposited in the GenBank given the accession no. OM063154. The strain was deposited at the Technology Innovation Center for Exploitation of Marine Biological Resources, Third Institute of Oceanography, Ministry of Natural Resources, China. For the fermentation, the fungus was activated on PDA medium (28 °C, 4 d), and then fresh mycelia and spores were inoculated into PDB medium under rotary culture (100 rpm, 25 °C, 3 d) to obtain seed cultures. Large-scale fermentation was carried out on rice solid medium in 1-L Erlenmeyer flasks, each containing 130 g of rice, 5.6 g of sea salt, and 170 mL of H_2_O. After autoclaving, each flask was inoculated with seed cultures (3 mL) and then incubated at 25 °C in static conditions for 50 days.

### 3.3. Extraction and Isolation

The fermented solid mash was extracted with ethyl acetate (EtOAc) four times to get a dark oily residue. The EtOAc extract was chromatographed on silica gel column CC eluting with petroleum ether (PE) and EtOAc (1:0–3:1) to yield five fractions (Fr. 1–Fr. 5). Fraction Fr. 2 was subjected to CC on ODS with a mobile phase of MeOH/H_2_O gradient elution (45–100%) to get 9 subfractions (SFr. 2-1–SFr. 2-9). Subfraction SFr. 2-7 was subjected to silica gel CC eluting with PE/EtOAc (3:1) and then with CH_2_Cl_2_/acetone (50:1) to yield six subfractions (SFr. 2-7-1–SFr. 2-7-6). Compounds **5** (84.8 mg) and **6** (2.5 mg) were isolated from the SFr. 2-7-4 by CC over semipreparative HPLC (CH_3_CN/H_2_O, 11:9, 2 mL/min) with a retention time (Rt) of 30 and 35 min, respectively. Fraction Fr. 4 was fractionated by the ODS CC eluted with MeOH in H_2_O (30–100%) and then over silica gel CC with CH_2_Cl_2_/MeOH gradient elution (100:1–10:1) to furnish six subfractions (SFr. 4-1–SFr. 4-6). Subfraction SFr. 4-3 was subjected to the semipreparative HPLC eluting with CH_3_CN/H_2_O (10:1, 2 mL/min) to yield **3** (6.5 mg) and **4** (23.5 mg) with the Rt of 25 and 18 min, respectively. Subfraction SFr. 4-5 was purified by Sephadex LH-20 eluting with MeOH and then by preparative HPLC (MeOH/H_2_O, 4:21, 2 mL/min) to obtain **1** (186.0 mg) and **2** (242.0 mg) with the Rt of 22 and 8.5 min, respectively.

Asperpropanol A (**1**): brownish red powder; [*α*${]}_{D}^{23}$ +3 (*c* 1.10, MeOH); UV (MeOH) *λ*_max_ (log *ε*) 214 (1.92), 288 (0.61) nm; ^1^H and ^13^C NMR data, Table 1 and Table 2; HRESIMS *m/z* 237.0727 [M + Na]^+^ (calcd for C_10_H_14_O_5_Na, 237.0739). IR (KBr) v_max_ 3425, 2928, 2930, 1603, 1395 cm^−1^.

Asperpropanol B (**2**): brownish red powder; [*α*${]}_{D}^{23}$ +5 (*c* 1.57, MeOH); UV (MeOH) *λ*_max_ (log *ε*) 214 (1.98), 288 (0.65) nm; ^1^H and ^13^C NMR data, Table 1 and Table 2; HRESIMS *m/z* 237.0739 [M + Na]^+^ (calcd for C_10_H_14_O_5_Na, 237.0739). IR (KBr) v_max_ 3414, 2983, 2937, 1610, 1475, 1387, 1344, 1265, 1000, 843 cm^−1^.

Asperpropanol C (**3**): yellow oil; [*α*${]}_{D}^{23}$ +73 (*c* 1.00, MeOH); UV (MeOH) *λ*_max_ (log *ε*) 213 (1.84), 292 (0.45) nm; ECD (MeOH) *λ*_max_ (Δ*ε*) 233 (−8.92), 279 (+19.03) nm; ^1^H and ^13^C NMR data, Table 1 and Table 2; HRESIMS *m/z* 235.0576 [M + Na]^+^ (calcd for C_10_H_12_O_5_Na, 235.0582). IR (KBr) v_max_ 3733, 3423, 2316, 1715, 1612, 1510, 1475, 1357, 1263, 996, 624 cm^−1^.

Asperpropanol D (**4**): yellow oil; [*α*${]}_{D}^{23}$ −4 (*c* 0.82, MeOH); UV (MeOH) *λ*_max_ (log *ε*) 214 (1.98), 288 (0.64) nm; ^1^H and ^13^C NMR data, Table 1 and Table 2; HRESIMS *m/z* 235.0571 [M + Na]^+^ (calcd for C_10_H_12_O_5_Na, 235.0582). IR (KBr) v_max_ 3734, 3427, 2983, 2930, 2316, 1684, 1610, 1467, 1394, 1270, 1067, 1003, 849 cm^−1^.

### 3.4. Preparation of (R)- and (S)-MPA Esters of **1**, **2**, and **4**

Compound **1** (1.5 mg) was dissolved in 500 μL of CHCl_3_. Then, reagent (*R*)-(-)-*α*-methoxyphenylacetic acid (*R*-MPA) (3.6 mg), N,N′-dicyclohexylcarbodiimide (DCC) (5.0 mg) and 4-dimethylaminopyridine (DMAP) (5.0 mg) were added. The mixture was stirred at room temperature for 12 h, and the reaction products were subjected to semipreparative HPLC eluting with 72% CH_3_CN in H_2_O to yield (*R*)-MPA ester of **1** (**1a**, 0.9 mg). The (*S*)-MPA ester of **1** (**1b**, 0.5 mg) was prepared similarly. Following the same protocol as for **1**, the (*R*)- and (*S*)-MPA esters of **2** and **4** were obtained, respectively.

(*R*)-MPA ester of **1** (**1a**): ^1^H NMR (CDCl_3_, 600 MHz) *δ*_H_ 5.92 (1H, d, *J* = 2.8 Hz, H-4), 6.53 (1H, d, *J* = 2.8 Hz, H-6), 6.00 (1H, d, *J* = 7.2 Hz, H-7), 5.16 (1H, m, H-8), 0.92 (3H, d, *J* = 6.5 Hz, H-9), 3.85 (3H, s, 2-OCH_3_).

(*S*)-MPA ester of **1** (**1b**): ^1^H NMR (CDCl_3_, 600 MHz) *δ*_H_ 6.48 (1H, d, *J* = 2.7 Hz, H-4), 6.58 (1H, d, *J* = 2.7 Hz, H-6), 6.07 (1H, d, *J* = 6.4 Hz, H-7), 5.18 (1H, m, H-8), 0.95 (3H, d, *J* = 6.5 Hz, H-9), 3.82 (3H, s, 2-OCH_3_).

(*R*)-MPA ester of **2** (**2a**): ^1^H NMR (CDCl_3_, 600 MHz) *δ*_H_ 6.53 (1H, d, *J* = 2.7 Hz, H-4), 6.61 (1H, d, *J* = 2.7 Hz, H-6), 6.25 (1H, d, *J* = 4.1 Hz, H-7), 5.12 (1H, m, H-8), 0.79 (3H, d, *J* = 6.5 Hz, H-9), 3.89 (3H, s, 2-OCH_3_).

(*S*)-MPA ester of **2** (**2b**): ^1^H NMR (CDCl_3_, 600 MHz) *δ*_H_ 6.01 (1H, d, *J* = 2.7 Hz, H-4), 6.47 (1H, d, *J* = 2.7 Hz, H-6), 6.02 (1H, d, *J* = 4.1 Hz, H-7), 5.21 (1H, m, H-8), 1.14 (3H, d, *J* = 6.5 Hz, H-9), 3.70 (3H, s, 2-OCH_3_).

(*R*)-MPA ester of **4** (**4a**): ^1^H NMR (CDCl_3_, 600 MHz) *δ*_H_ 6.68 (1H, d, *J* = 3.0 Hz, H-4), 6.61 (1H, d, *J* = 3.0 Hz, H-6), 5.98 (1H, q, *J* = 7.1 Hz, H-8), 1.37 (3H, d, *J* = 7.2 Hz, H-9), 3.84 (3H, s, 2-OCH_3_).

(*S*)-MPA ester of **4** (**4b**): ^1^H NMR (CDCl_3_, 600 MHz) *δ*_H_ 6.62 (1H, d, *J* = 3.0 Hz, H-4), 6.49 (1H, d, *J* = 3.0 Hz, H-6), 5.99 (1H, q, *J* = 7.1 Hz, H-8), 1.46 (3H, d, *J* = 7.1 Hz, H-9), 3.76 (3H, s, 2-OCH_3_).

### 3.5. Cell Culture

Raw264.7 was purchased from the Cell Bank of Chinese Academy of Sciences (Catalog number: SCSP-5036, Shanghai, China) and cultured in DMEM (Gibco, Carlsbad, CA, USA) containing 10% fetal bovine serum (FBS; Gibco). Exponentially growing cells were used for further experiments.

### 3.6. Cell Viability

Raw264.7 cells were seeded in a 96-well plate at the intensity of 1 × 10^4^ cells per well and cultured overnight. Then, the compounds (10 mM stock solutions, dissolved in DMSO) were added at the working concentration of 20 μM and cultured for 24 h (*n* = 6). After that, the original DMEM were discarded, and 100 μL of new culture medium with 10% Cell Counting Kit-8 (CCK8, MCE, Monmouth Junction, NJ, USA) was added in each well and cultured for 2 h. The absorbance at 450 nm was measured with a microplate reader (Tecan Sunrise, TECAN Deutschland GmbH, Crailsheim, Germany), and cell viability was calculated according to the manufacturer’s instructions.

### 3.7. Nitrite Determination

Raw264.7 cells were seeded in a 96-well plate at 1 × 10^4^ cells/well and treated with vehicle (normal control, NC), 1 μg/mL LPS (L4391, Sigma-Aldrich, St. Louis, MO, USA), LPS combined with 10 μM of BAY11-7083 (BAY, Selleck, Huston, USA), LPS combined with the compounds (20 μM), or LPS combined with the compounds and 5 μM of ML385 (Selleck). The cells were treated for 24 h, and the nitrite in the culture medium was determined with Griess reagent (Thermo Fisher Scientific, Shanghai, China) according to the manufacturer’s protocol.

### 3.8. Enzyme-Linked Immunosorbent Assays

Cytokine levels in the cell culture medium were determined with corresponding kits (EK282/4-96 for TNF-α and EK206/3-96 for IL-6, Multi Sciences, Hangzhou, China) according to the manufacturer’s protocols.

### 3.9. Quantitative RT-PCR Analysis

Total RNA of Raw264.7 cells was extracted using Trizol reagent (Biouniquer Technology Co., Ltd., Nanjing, China) and converted to cDNA using a commercial kit (R333-01, Vazyme Biotech, Nanjing, China). Quantification of gene expression was performed on the LightCycler 96 instrument (Roche, Basel, Switzerland) using Taq Pro Universal SYBR qPCR Master Mix (Q712-02, Vazyme Biotech) according to the manufacturer’s instructions. The 2^−ΔΔCt^ methods were used to measure relative transcript mRNA level of iNOS, COX-2, and Nrf2. β-actin was used as the invariant control. The primers used are listed in Table 3.

## 4. Conclusions

Asperpropanols A–D (**1**–**4**), four undescribed phenolic compounds, along with two known analogous **5** and **6**, were isolated from a deep-sea-derived fungus *Aspergillus puniceus* A2. Their structures, including the absolute configurations, were established by detailed analyses of the HRESIMS and NMR data, modified Mosher’s method and calculations of ECD spectra. Furthermore, **1**–**6** exerted their anti-inflammatory effects probably via Nrf2 upregulation and activation.

## Figures and Tables

**Table 1:** ^1^H NMR data of **1**–**4** measured in DMSO-*d*_6_ at 600 MHz (*δ* in ppm, *J* in Hz).

No.	1	2	3	4
4	6.20, br s	6.16, d (2.8)	6.27, d (2.9)	6.48, d (2.8)
6	6.20, br s	6.27, d (2.8)	6.12, d (2.9)	6.25, d (2.8)
7	4.48, dd (6.5, 3.4)	4.65, t (4.4)	5.10, d (4.7)	
8	3.60, m	3.68, m		4.76, quint (6.6)
9	0.87, d (6.4)	0.95, d (6.3)	2.03, s	1.17, d (6.9)
2-OCH_3_	3.62, s	3.61, s	3.63, s	3.66, s
3-OH	9.07, s	8.97, s	9.27, s	9.58, s
5-OH	8.86, s	8.79, s	9.03, s	9.30, s
7-OH	4.94, d (3.4)	4.79, d (4.4)	5.59, d (4.7)	
8-OH	4.49, d (4.2)	4.36, d (5.5)		5.08, d (6.2)

**Table 2:** ^13^C NMR spectroscopic data of **1**–**4** measured in DMSO-*d*_6_ at 150 MHz.

No.	1	2	3	4
1	137.2, C	137.3, C	134.2, C	132.8, C
2	138.3, C	138.2, C	138.5, C	139.0, C
3	150.4, C	150.2, C	151.0, C	151.5, C
4	103.0, CH	102.8, CH	104.1, CH	107.4, CH
5	153.6, C	153.4, C	153.9, C	153.9, C
6	104.7, CH	104.7, CH	105.2, CH	105.0, CH
7	72.3, CH	71.5, CH	74.7, CH	205.6, C
8	71.2, CH	69.9, CH	208.3, C	71.8, CH
9	19.8, CH_3_	17.9, CH_3_	26.0, CH_3_	20.4, CH_3_
2-OMe	60.5, CH_3_	60.4, CH_3_	60.6, CH_3_	61.4, CH_3_

**Table 3:** Primers used for RT-PCR.

Genes	Forward Primer	Reverse Primer
iNOS	CGGCAAACATGACTTCAGGC	TCGATGCACAACTGGGTGAA
COX-2	GCTGGAAAAGGTTCTTCTACG	AACCCAGGTCCTCGCTTA
Nrf2	TCAGCGACAGAAGGACTAAG	AGGCATCTTGTTTGGAATG
β-actin	AGCCATGTACGTAGCCATCC	CTCTCAGCTGTGGTGGTGAA

## Data Availability

All data supporting the conclusions of this article are included in this article.

## Supplementary Materials

The following supporting information can be downloaded at: https://www.mdpi.com/article/10.3390/md20090575/s1.

## References

[1] Zhao W.-Y., Yi J., Chang Y.-B., Sun C.-P., Ma X.-C. (2022). Recent studies on terpenoids in *Aspergillus fungi*: Chemical diversity, biosynthesis, and bioactivity. Phytochemistry.

[2] Frisvad J.C., Larsen T.O. (2015). Chemodiversity in the genus *Aspergillus*. Appl. Microbiol. Biotechnol..

[3] Alberts A., Chen J., Kuron G., Hunt V., Huff J., Hoffman C., Rothrock J., Lopez M., Joshua H., Harris E. (1980). Mevinolin: A highly potent competitive inhibitor of hydroxymethylglutaryl-coenzyme A reductase and a cholesterol-lowering agent. Proc. Natl. Acad. Sci. USA.

[4] Numata A., Takahashi C., Matsushita T., Miyamoto T., Kawai K., Usami Y., Matsumura E., Inoue M., Ohishi H., Shingu T. (1992). Fumiquinazolines, novel metabolites of a fungus isolated from a saltfish. Tetrahedron Lett..

[5] Ma L., Tian X., Li G., Zhao Y., Yin J. (2021). Research status and development trends of natural products from marine microorganisms. J. Trop. Oceanogr..

[6] Liang X., Zhang X., Lu X., Zheng Z., Ma X., Qi S. (2019). Diketopiperazine-type alkaloids from a deep-sea-derived *Aspergillus puniceus fungus* and their effects on liver X receptor α. J. Nat. Prod..

[7] Liang X., Huang Z.-H., Ma X., Zheng Z.-H., Zhang X.-X., Lu X.-H., Qi S.-H. (2021). Mycotoxins as inhibitors of protein tyrosine phosphatases from the deep-sea-derived fungus *Aspergillus puniceus* SCSIO z021. Bioorg. Chem..

[8] Liu C.-M., Yao F.-H., Lu X.-H., Zhang X.-X., Luo L.-X., Liang X., Qi S.-H. (2022). Isoquinoline Alkaloids as Protein Tyrosine Phosphatase Inhibitors from a Deep-Sea-Derived Fungus *Aspergillus puniceus*. Mar. Drugs.

[9] Chen L., Deng H., Cui H., Fang J., Zuo Z., Deng J., Li Y., Wang X., Zhao L. (2018). Inflammatory responses and inflammation-associated diseases in organs. Oncotarget.

[10] Furman D., Campisi J., Verdin E., Carrera-Bastos P., Targ S., Franceschi C., Ferrucci L., Gilroy D.W., Fasano A., Miller G.W. (2019). Chronic inflammation in the etiology of disease across the life span. Nat. Med..

[11] Freire F., Seco J.M., Quiñoá E., Riguera R. (2005). Determining the absolute stereochemistry of secondary/secondary diols by ^1^H NMR: Basis and applications. J. Org. Chem..

[12] Frisch M.J.T., Trucks G.W., Schlegel H.B., Scuseria G.E., Robb M.A., Cheeseman J.R., Scalmani G., Barone V., Mennucci B., Petersson G.A. (2009). Gaussian 09.

[13] Saepua S., Kornsakulkarn J., Somyong W., Laksanacharoen P., Isaka M., Thongpanchang C. (2018). Bioactive compounds from the scale insect fungus *Conoideocrella tenuis* BCC 44534. Tetrahedron.

[14] Latypov S.K., Seco J.M., Quiñoá E., Riguera R. (1996). MTPA vs MPA in the determination of the absolute configuration of chiral alcohols by ^1^H NMR. J. Org. Chem..

[15] Sun W., Zhuang C., Li X., Zhang B., Lu X., Zheng Z., Dong Y. (2017). Varic acid analogues from fungus as PTP1B inhibitors: Biological evaluation and structure-activity relationships. Bioorg. Med. Chem. Lett..

[16] Wen H., Chen C., Sun W., Zang Y., Li Q., Wang W., Zeng F., Liu J., Zhou Y., Zhou Q. (2019). Phenolic C-Glycosides and Aglycones from Marine-Derived *Aspergillus* sp. and Their Anti-Inflammatory Activities. J. Nat. Prod..

[17] Oates J.C., Gilkeson G.S. (2006). The biology of nitric oxide and other reactive intermediates in systemic lupus erythematosus. Clin. Immunol..

[18] Hinson R.M., Williams J.A., Shacter E. (1996). Elevated interleukin 6 is induced by prostaglandin E2 in a murine model of inflammation: Possible role of cyclooxygenase-2. Proc. Natl. Acad. Sci. USA.

[19] Anderson G.D., Hauser S.D., McGarity K.L., Bremer M.E., Isakson P.C., Gregory S.A. (1996). Selective inhibition of cyclooxygenase (COX)-2 reverses inflammation and expression of COX-2 and interleukin 6 in rat adjuvant arthritis. J. Clin. Investig..

[20] Zhao Y., Usatyuk P.V., Gorshkova I.A., He D., Wang T., Moreno-Vinasco L., Geyh A.S., Breysse P.N., Samet J.M., Spannhake E.W. (2009). Regulation of COX-2 expression and IL-6 release by particulate matter in airway epithelial cells. Am. J. Respir. Cell Mol. Biol..

[21] Nakao S., Ogtata Y., Shimizu E., Yamazaki M., Furuyama S., Sugiya H. (2002). Tumor necrosis factor α (TNF-α)-induced prostaglanding E2 release is mediated by the activation of cyclooxygenase-2 (COX-2) transcription via NFκB in human gingival fibroblasts. Mol. Cell. Biochem..

[22] Young W., Mahboubi K., Haider A., Li I., Ferreri N.R. (2000). Cyclooxygenase-2 is required for tumor necrosis factor-α–and angiotensin II–mediated proliferation of vascular smooth muscle cells. Circ. Res..

[23] Vomund S., Schäfer A., Parnham M.J., Brüne B., Von Knethen A. (2017). Nrf2, the master regulator of anti-oxidative responses. Int. J. Mol. Sci..

[24] Petri S., Körner S., Kiaei M. (2012). Nrf2/ARE signaling pathway: Key mediator in oxidative stress and potential therapeutic target in ALS. Neurol. Res. Int..

[25] Robledinos-Antón N., Fernández-Ginés R., Manda G., Cuadrado A. (2019). Activators and inhibitors of NRF2: A review of their potential for clinical development. Oxid. Med. Cell. Longev..

