# Supplementary material for: Phenolic Metabolites from a Deep-Sea-Derived Fungus Aspergillus puniceus A2 and Their Nrf2-Dependent Anti-Inflammatory Effects
Source: Mar Drugs. 2022 Sep 13;20(9):575. doi: 10.3390/md20090575 (PMC9505415; doi:10.3390/md20090575)
Supplement: Supplementary file 1 [file marinedrugs-20-00575-s001.zip › marinedrugs-1896570-supplementary.pdf]

Supporting Information

Phenolic metabolites from a deep-sea-derived fungus *Aspergillus puniceus* A2 and their Nrf2-dependent anti-inflammatory effects

Jianlin He[1, 2, 3], Xin Wu[1], Shuhuan Huang[1, 2, 3], Juan Wang[1, 2, 3], SiwenNiu[1, 2, 3], Meixiang Chen[1, 2, 3], Gaiyun Zhang[1], Songyan Cai[1, 2, 3], Jingna Wu[4] and Bihong Hong[1, 2, 3]*

1 Third Institute of Oceanography, Ministry of Natural Resources, Xiamen 361005, PR China.

2 Technical Innovation Center for Exploitation of Marine Biological Resources, Ministry of Natural Resources, Xiamen 361005, PR China.

3 Fujian Provincial Key Laboratory of Island Conservation and Development (Island Research Center, MNR), Pingtan 350400, China

4 Xiamen Medical College, Xiamen 361023, PR China

* Correspondence: bhhong@tio.org.cn; Tel.: (+86) 0592-2195265

Contents Pages

Figure S1. HRESIMS spectrum of **1**. S4

Figure S2. ^{1}H NMR spectrum of **1** in DMSO-d_6 (600 MHz). S5

Figure S3. ^{13}C NMR spectrum of **1** in DMSO-d_6 (150 MHz). S6

Figure S4. HSQC spectrum of **1** in DMSO-d_6. S7

Figure S5. COSY spectrum of **1** in DMSO-d_6. S8

Figure S6. HMBC spectrum of **1** in DMSO-d_6. S9

Figure S7. ^{1}H NMR spectrum of (*R*)-MPA ester of **1** (**1a**) in $CDCl_3$ at 600 MHz. S10

Figure S8. ^{1}H NMR spectrum of (*S*)-MPA ester of **1** (**1b**) in $CDCl_3$ at 600 MHz. S11

Figure S9. IR spectrum of **1**. S12

Figure S10. HRESIMS spectrum of **2**. S13

Figure S11. ^{1}H NMR spectrum of **2** in DMSO-d_6 (600 MHz). S14

Figure S12. ^{13}C NMR spectrum of **2** in DMSO-d_6 (150 MHz). S15

Figure S13. HSQC spectrum of **2** in DMSO-d_6. S16

Figure S14. COSY spectrum of **2** in DMSO-d_6. S17

Figure S15. HMBC spectrum of **2** in DMSO-d_6. S18

Figure S16. ^{1}H NMR spectrum of (*R*)-MPA ester of **2** (**2a**) in $CDCl_3$ at 600 MHz. S19

Figure S17. ^{1}H NMR spectrum of (*S*)-MPA ester of **2** (**2b**) in $CDCl_3$ at 600 MHz. S20

Figure S18. IR spectrum of **2**. S21

Figure S19. HRESIMS spectrum of **3**. S22

Figure S20. ^{1}H NMR spectrum of **3** in DMSO-d_6 (600 MHz). S23

Figure S21. ^{13}C NMR spectrum of **3** in DMSO-d_6 (150 MHz). S24

Figure S22. HSQC spectrum of **3** in DMSO-d_6. S25

Figure S23. COSY spectrum of **3** in DMSO-d_6. S26

Figure S24. HMBC spectrum of **3** in DMSO-d_6. S27

Figure S25. IR spectrum of **3**. S28

Figure S26. HRESIMS spectrum of **4**. S29

Figure S27. ^{1}H NMR spectrum of **4** in DMSO-d_6 (600 MHz). S30

Figure S28. ^{13}C NMR spectrum of **4** in DMSO-d_6 (150 MHz). S31

Figure S29. HSQC spectrum of **4** in DMSO-d_6. S32

Figure S30. COSY spectrum of **4** in DMSO-d_6. S33

Figure S31. HMBC spectrum of **4** in DMSO-d_6. S34

Figure S32. ^{1}H NMR spectrum of (*R*)-MPA ester of **4** (**4a**) in $CDCl_3$ at 600 MHz. S35

Figure S33. ^{1}H NMR spectrum of (*S*)-MPA ester of **4** (**4b**) in $CDCl_3$ at 600 MHz. S36

Figure S34. IR spectrum of **4**. S37

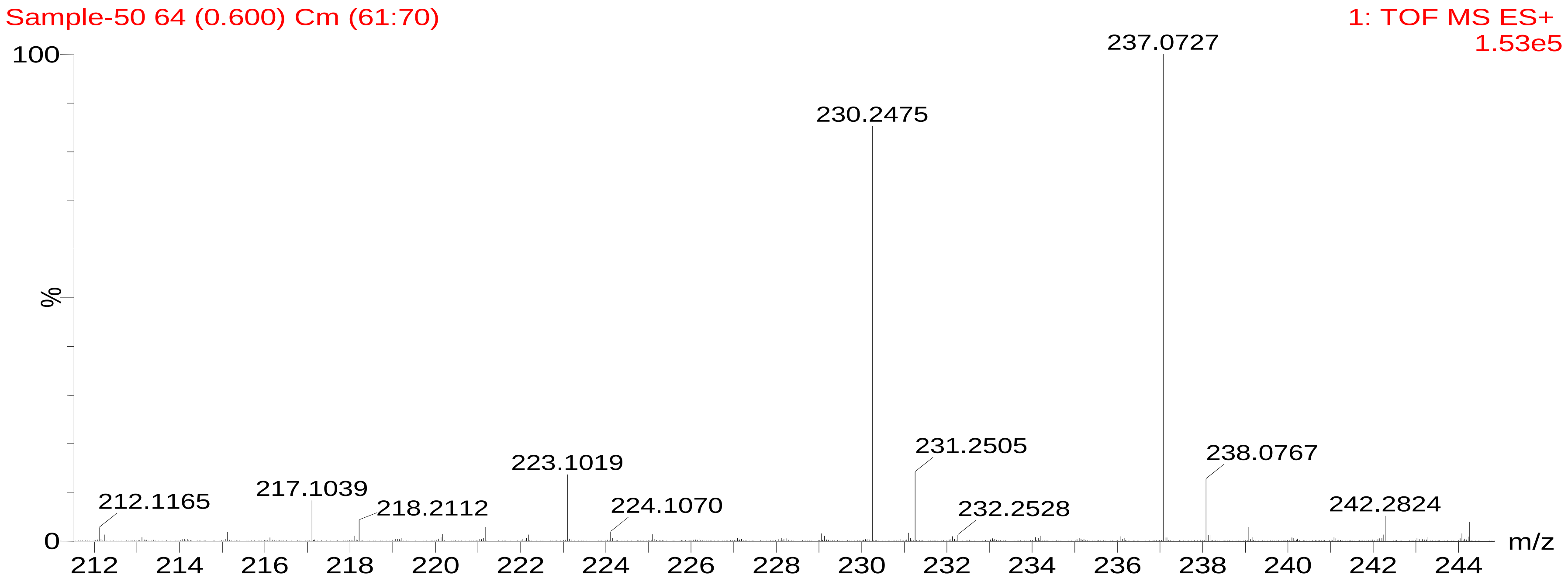

Figure S1. HRESIMS spectrum of **1**.

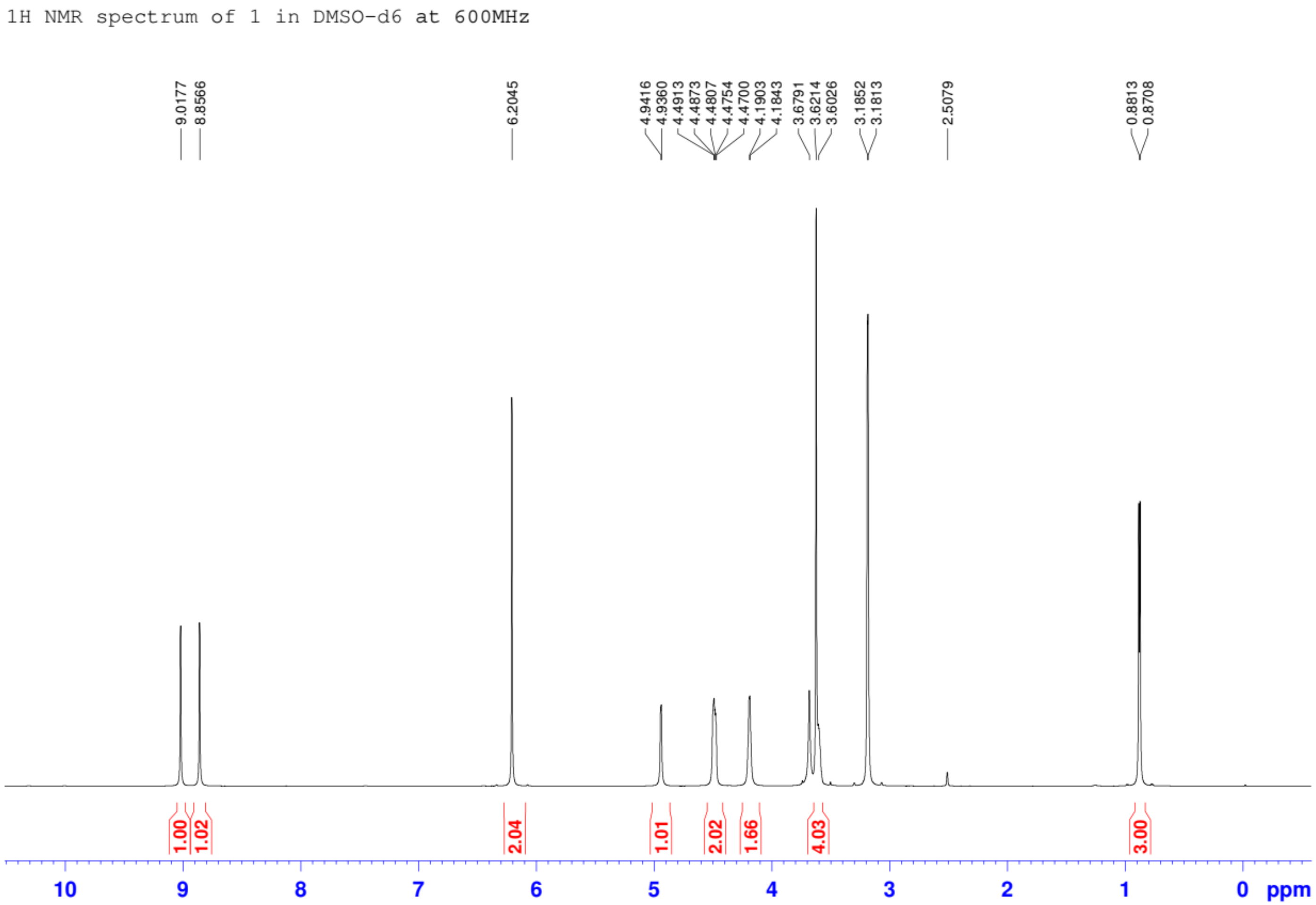

Figure S2. ^{1}H NMR spectrum of **1** in DMSO-d_6 (600 MHz).

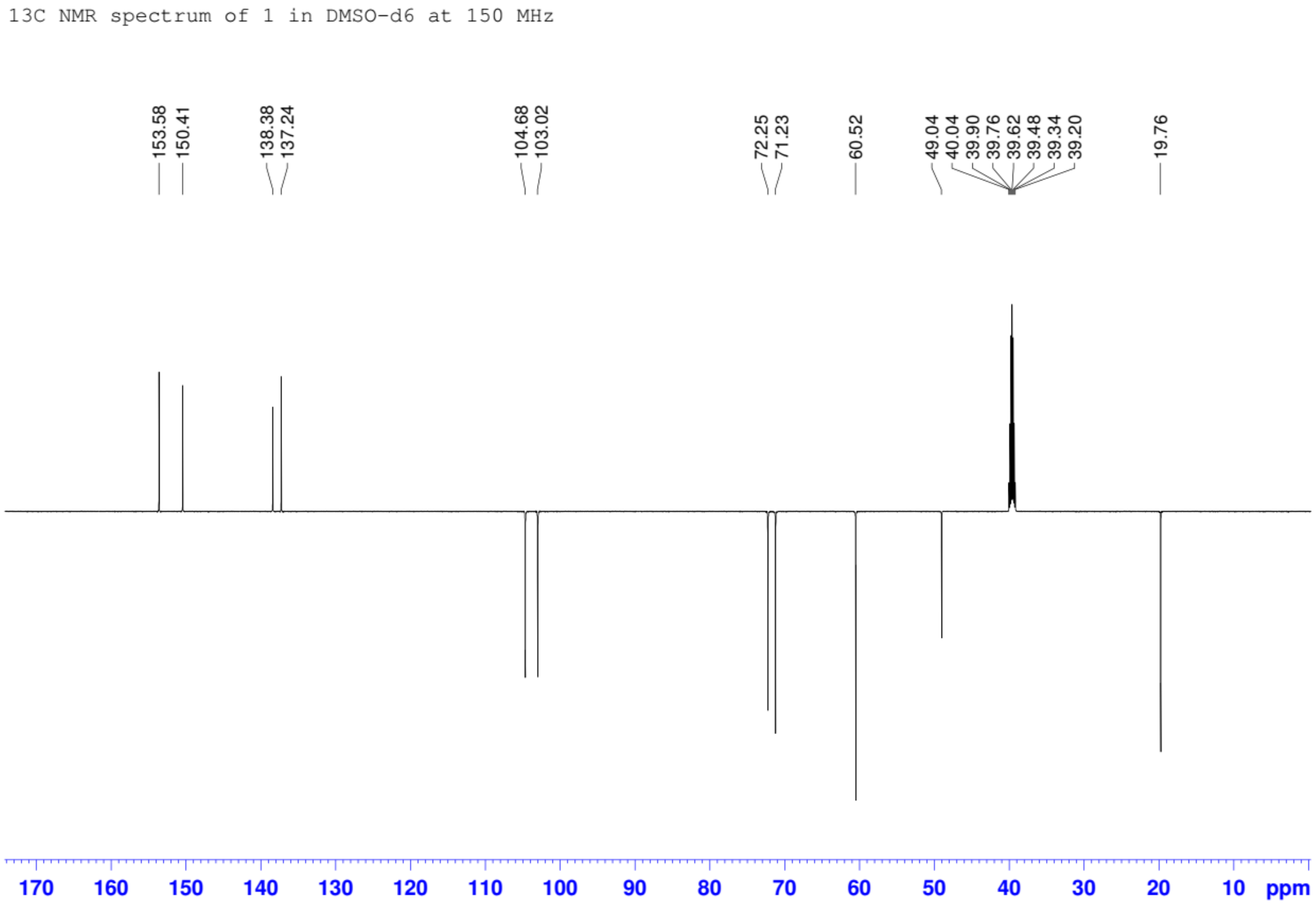

Figure S3. ^{13}C NMR spectrum of **1** in DMSO-d_6 (150 MHz).

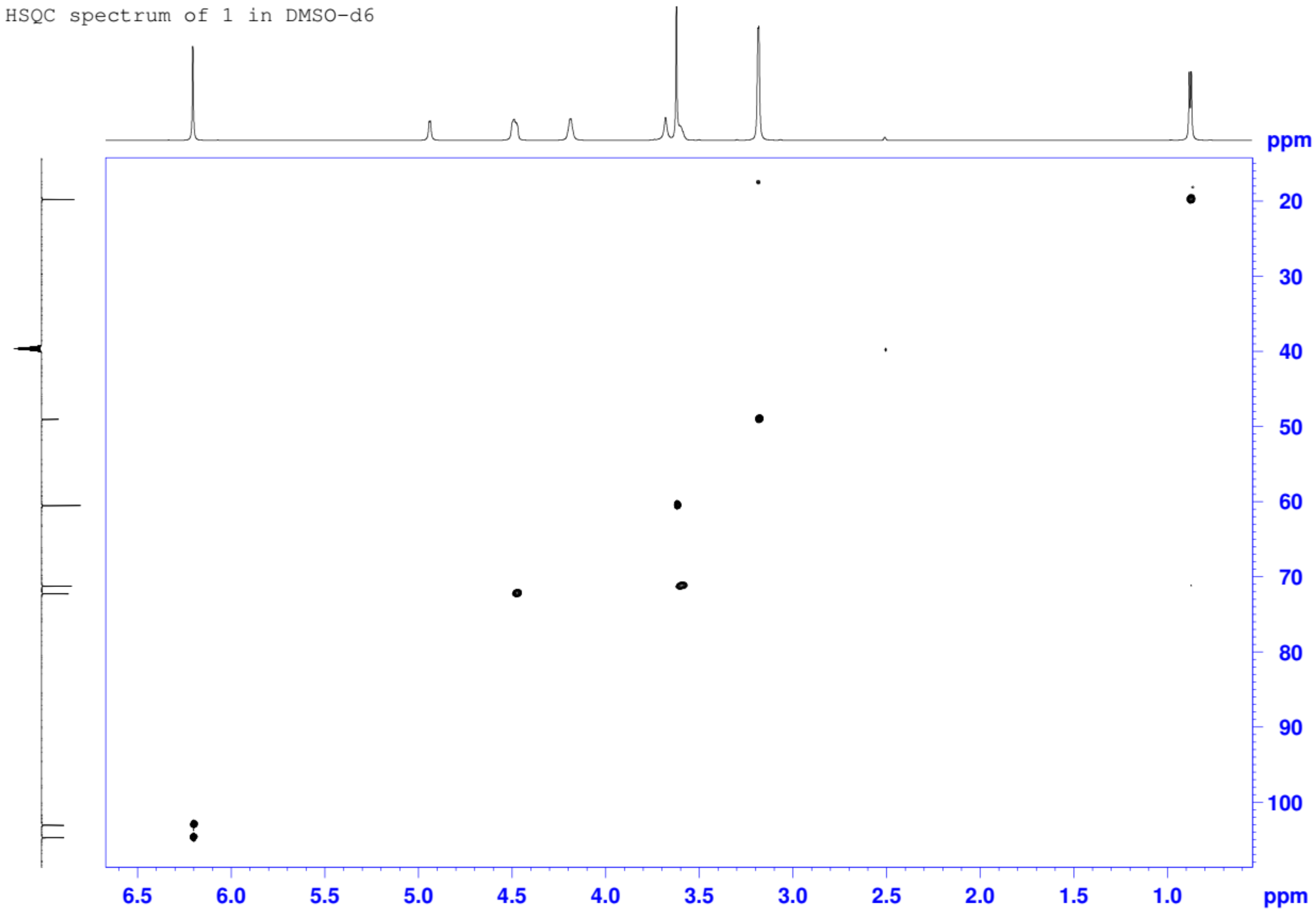

Figure S4. HSQC spectrum of **1** in DMSO-d_6.

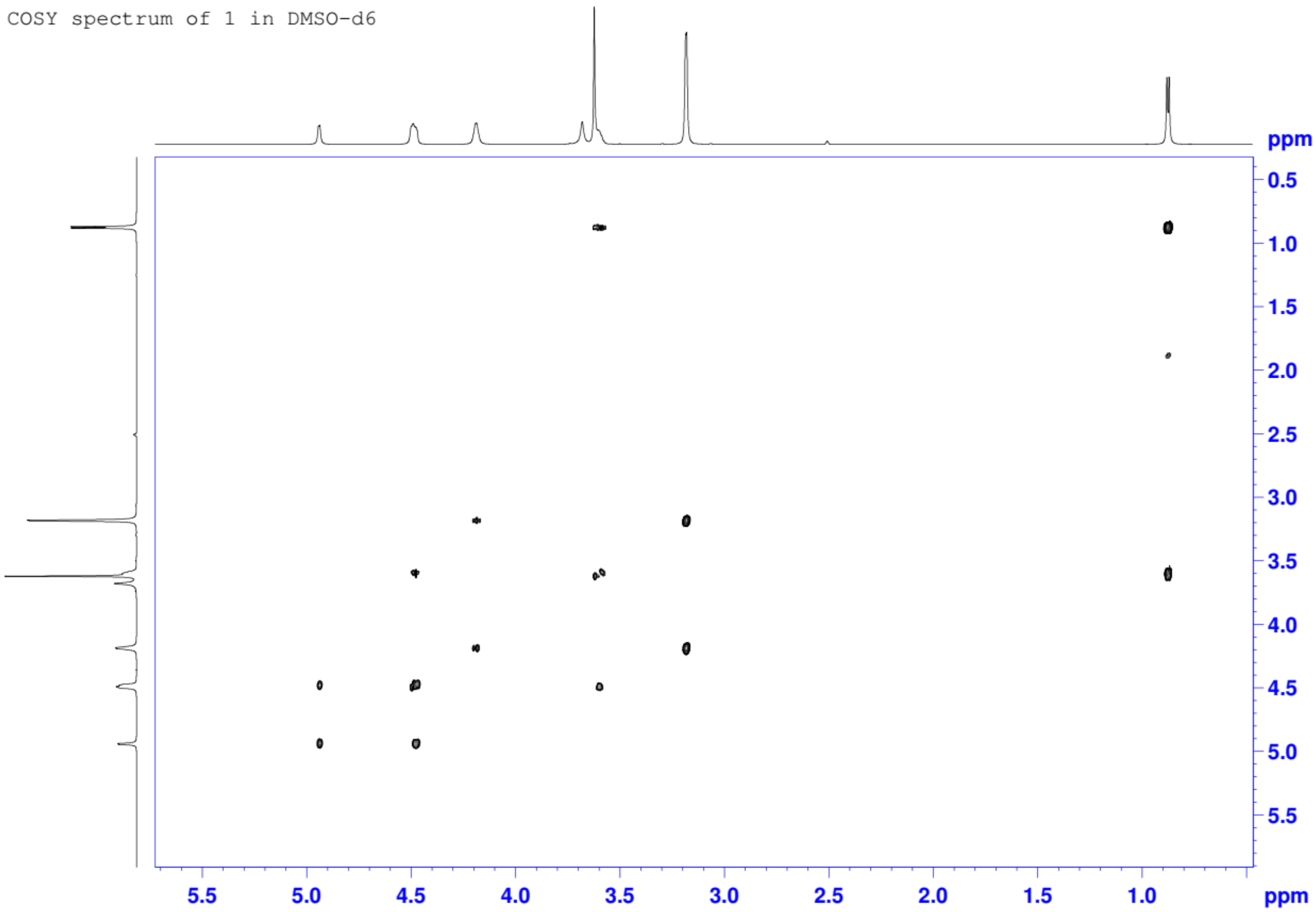

Figure S5. COSY spectrum of **1** in DMSO-d_6.

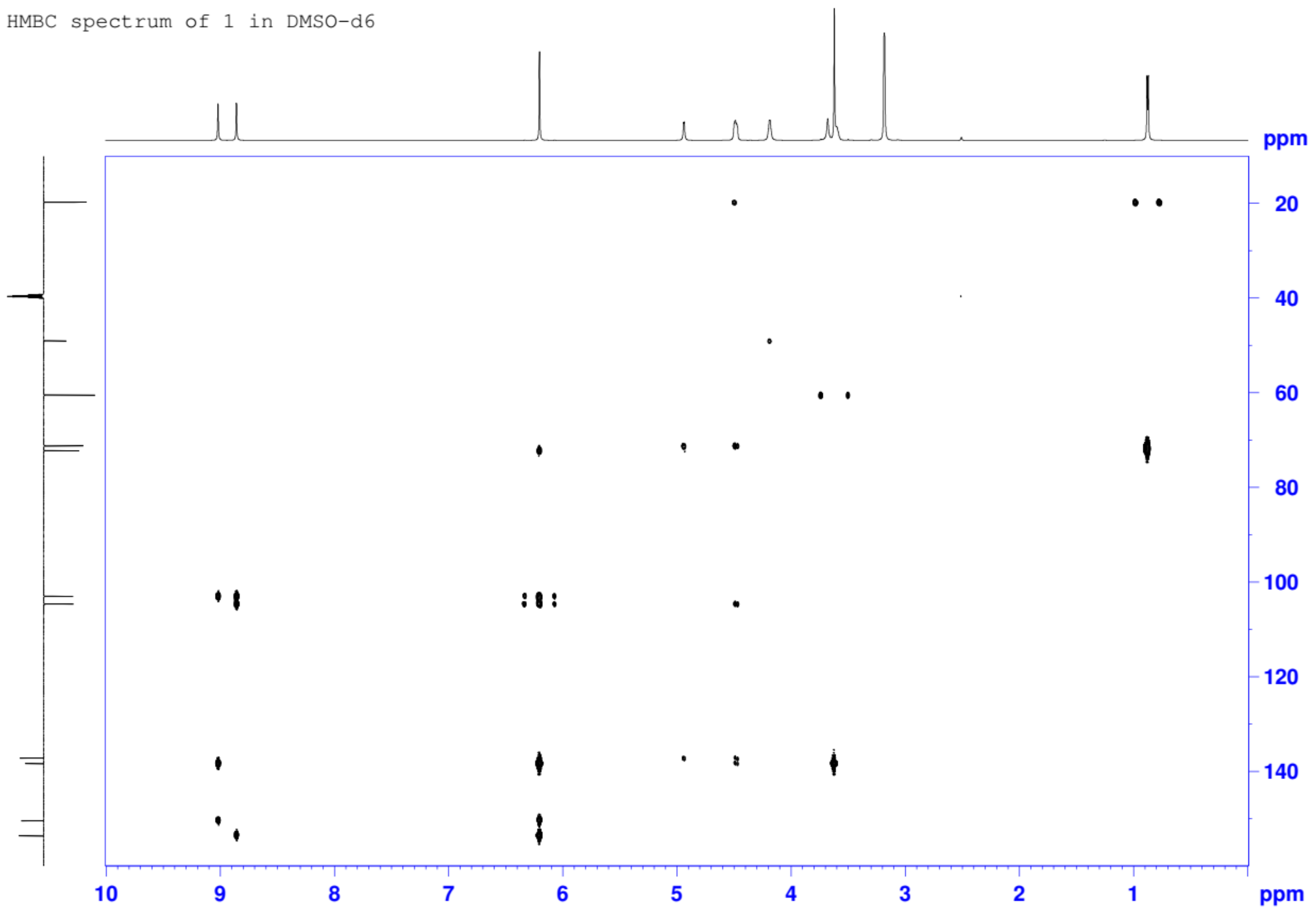

Figure S6. HMBC spectrum of **1** in DMSO-d_6.

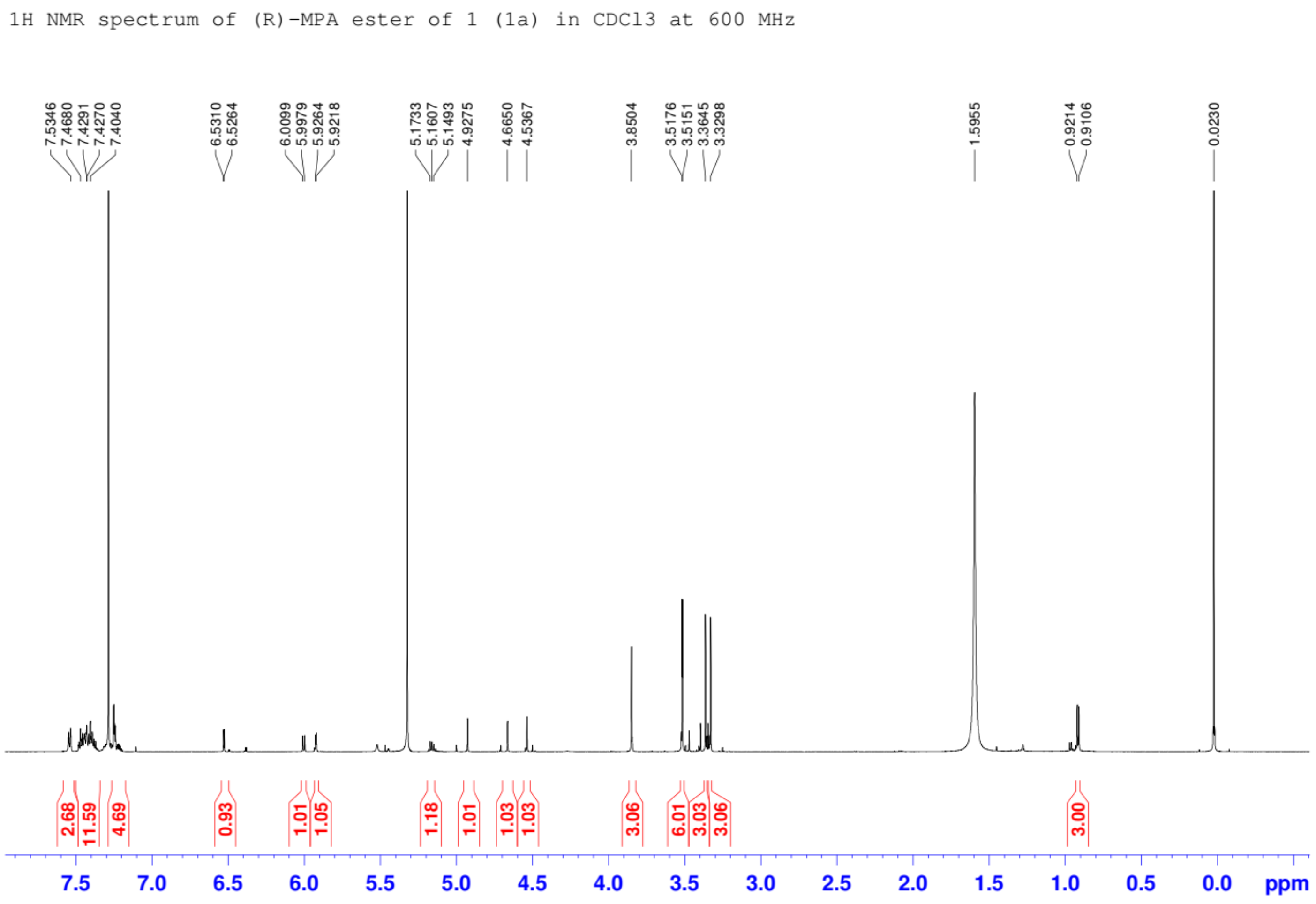

Figure S7. ^{1}H NMR spectrum of (*R*)-MPA ester of **1** (**1a**) in $CDCl_3$ at 600 MHz.

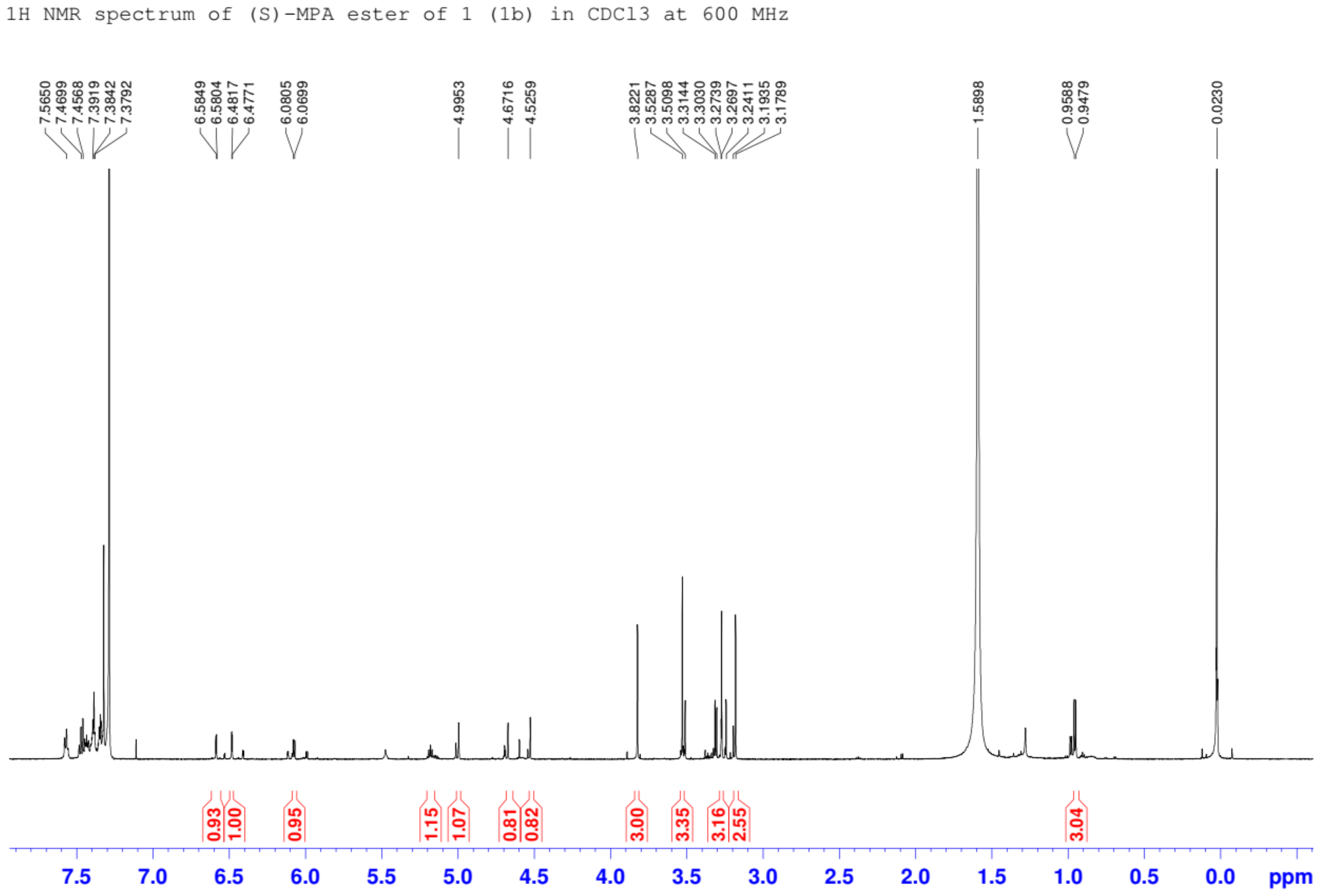

Figure S8. ^{1}H NMR spectrum of (*S*)-MPA ester of **1** (**1b**) in $CDCl_3$ at 600 MHz.

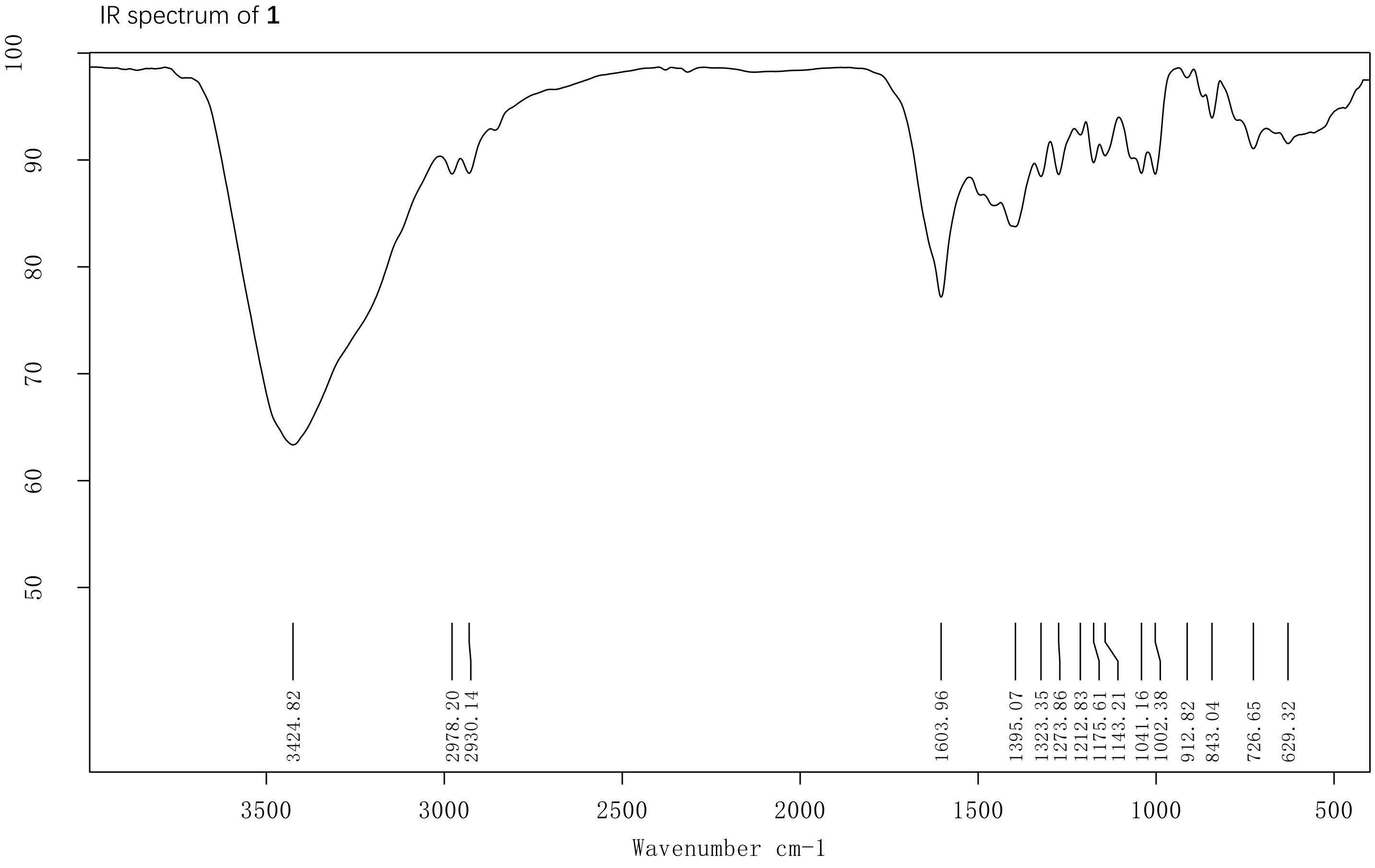

Figure S9. IR spectrum of **1**.

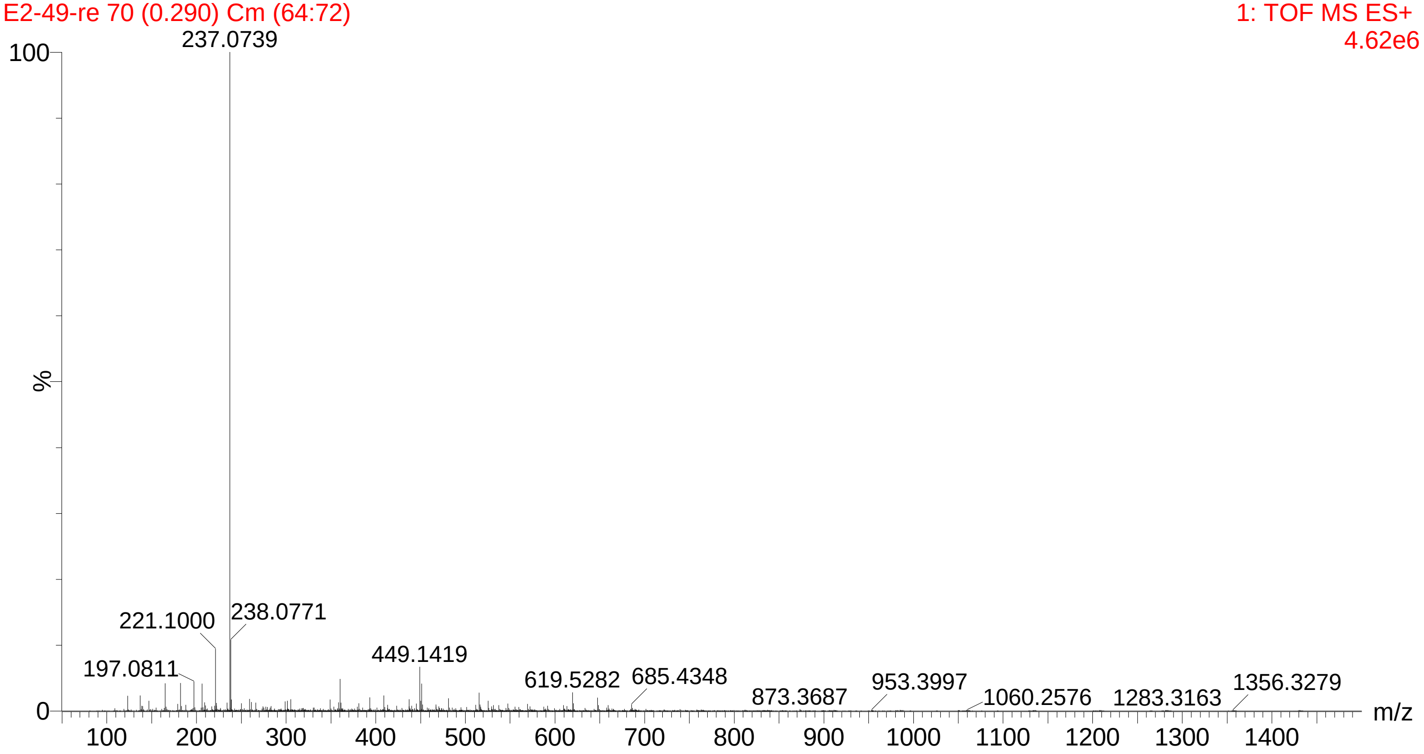

Figure S10. HRESIMS spectrum of **2**.

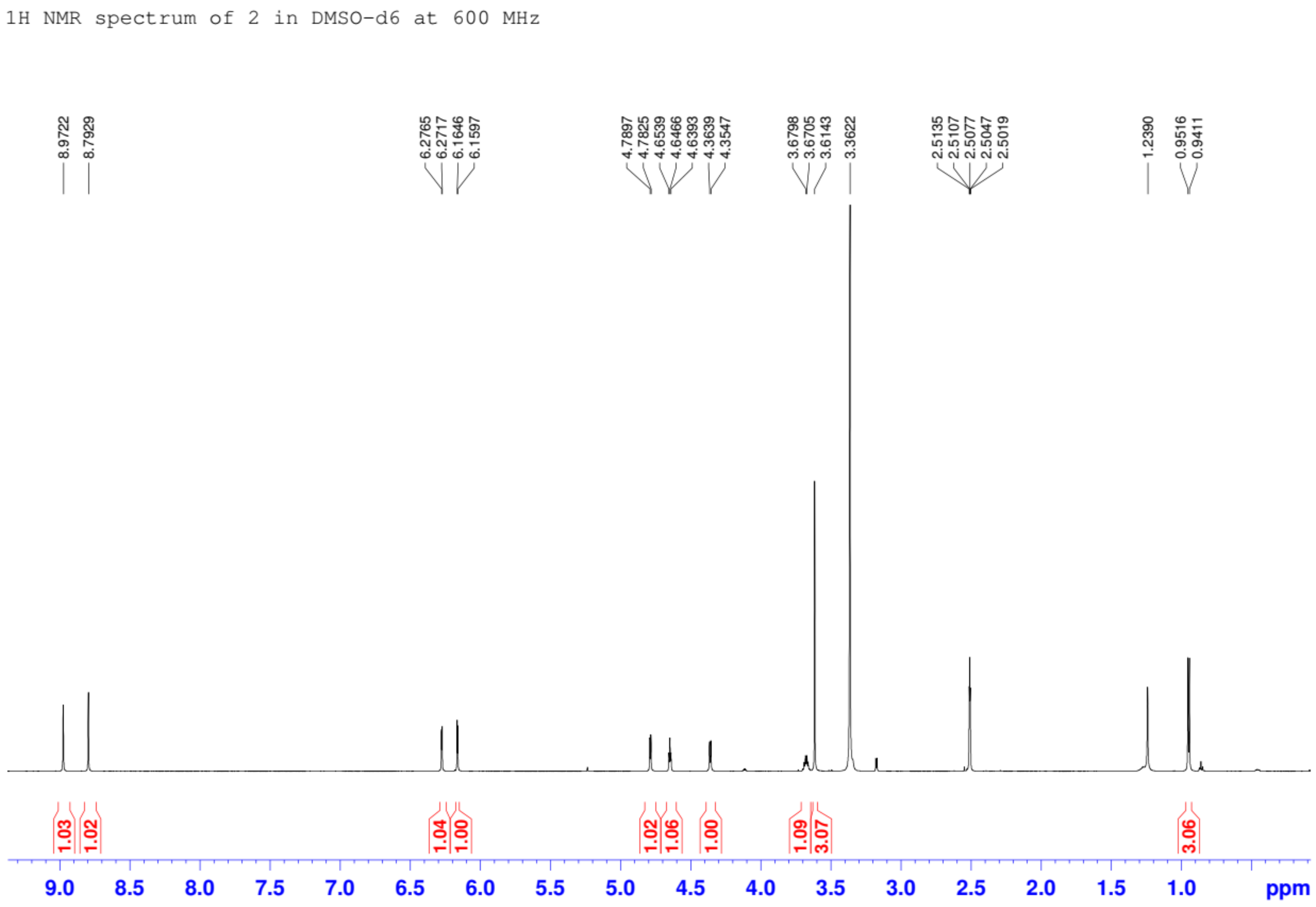

Figure S11. ^{1}H NMR spectrum of **2** in DMSO-d_6 (600 MHz).

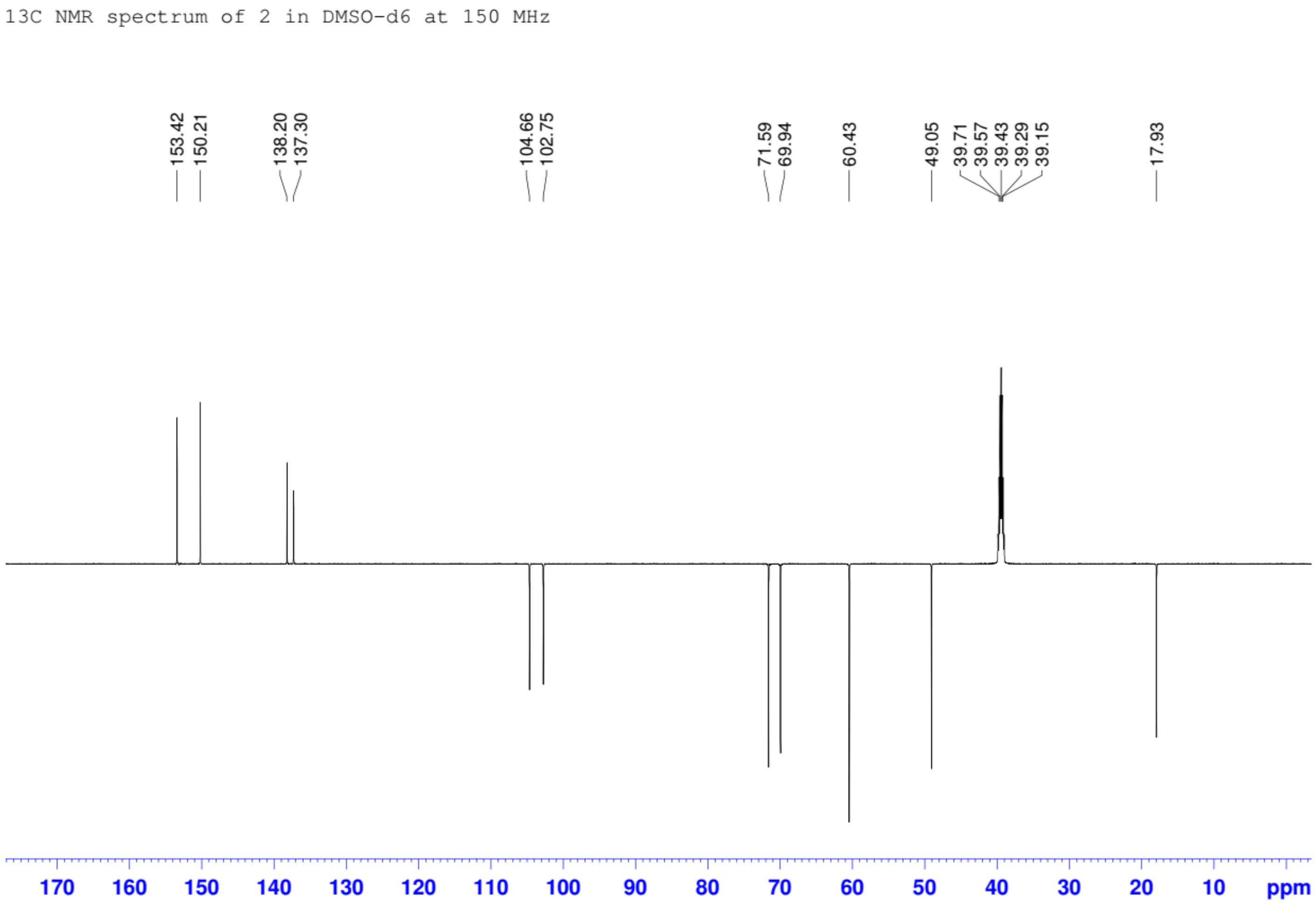

Figure S12. ^{13}C NMR spectrum of **2** in DMSO-d_6 (150 MHz).

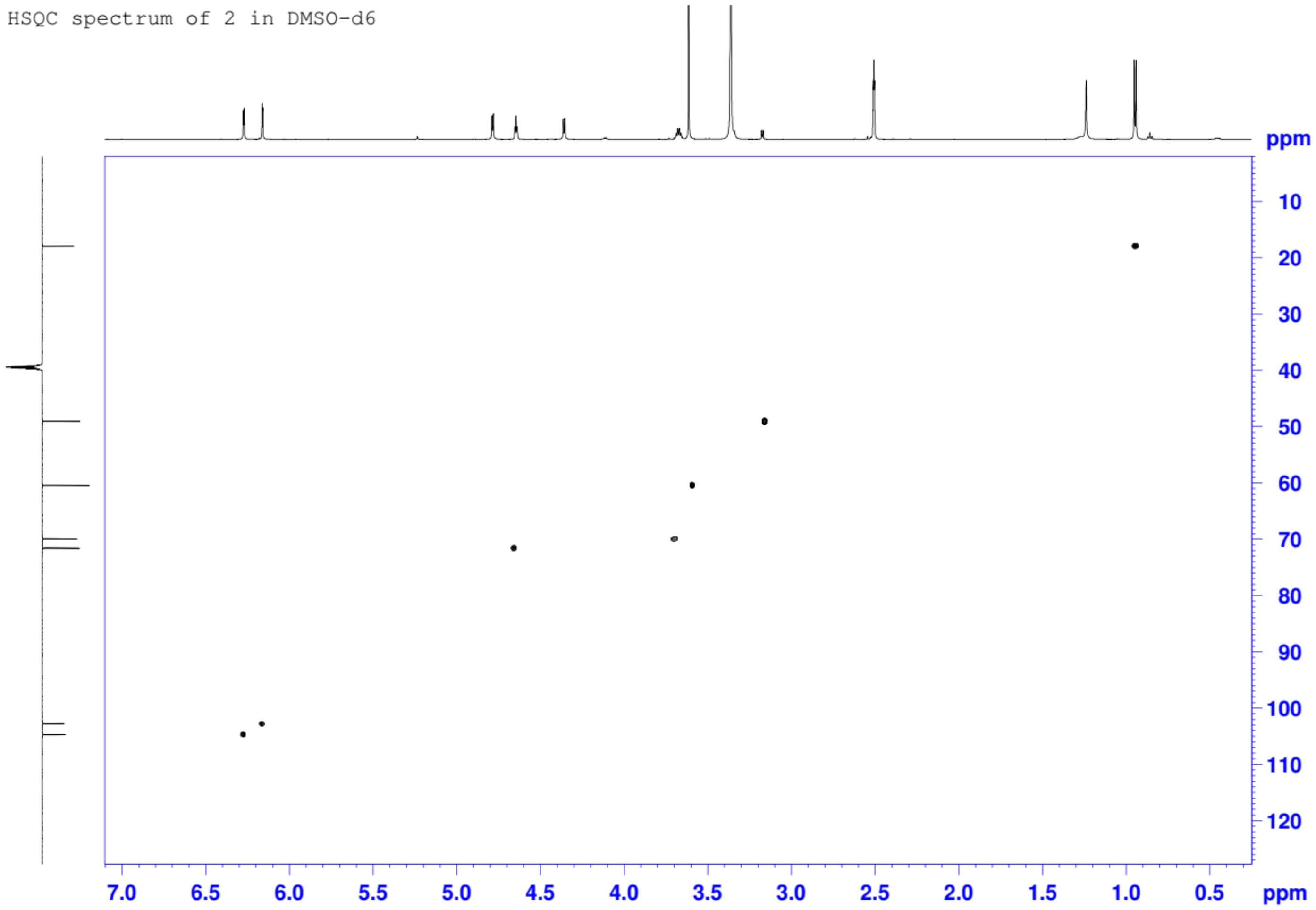

Figure S13. HSQC spectrum of **2** in DMSO-d_6.

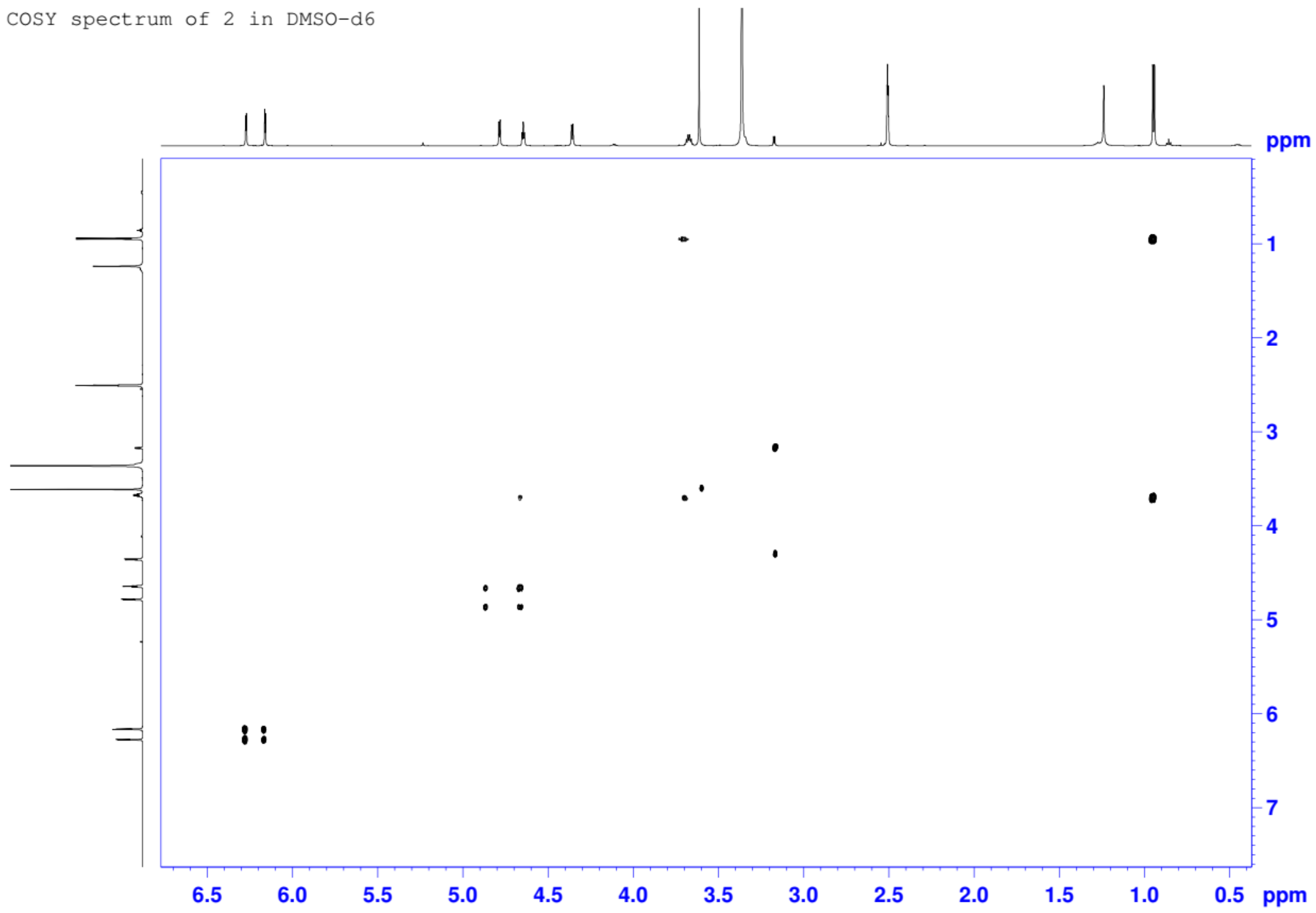

Figure S14. COSY spectrum of **2** in DMSO-d_6.

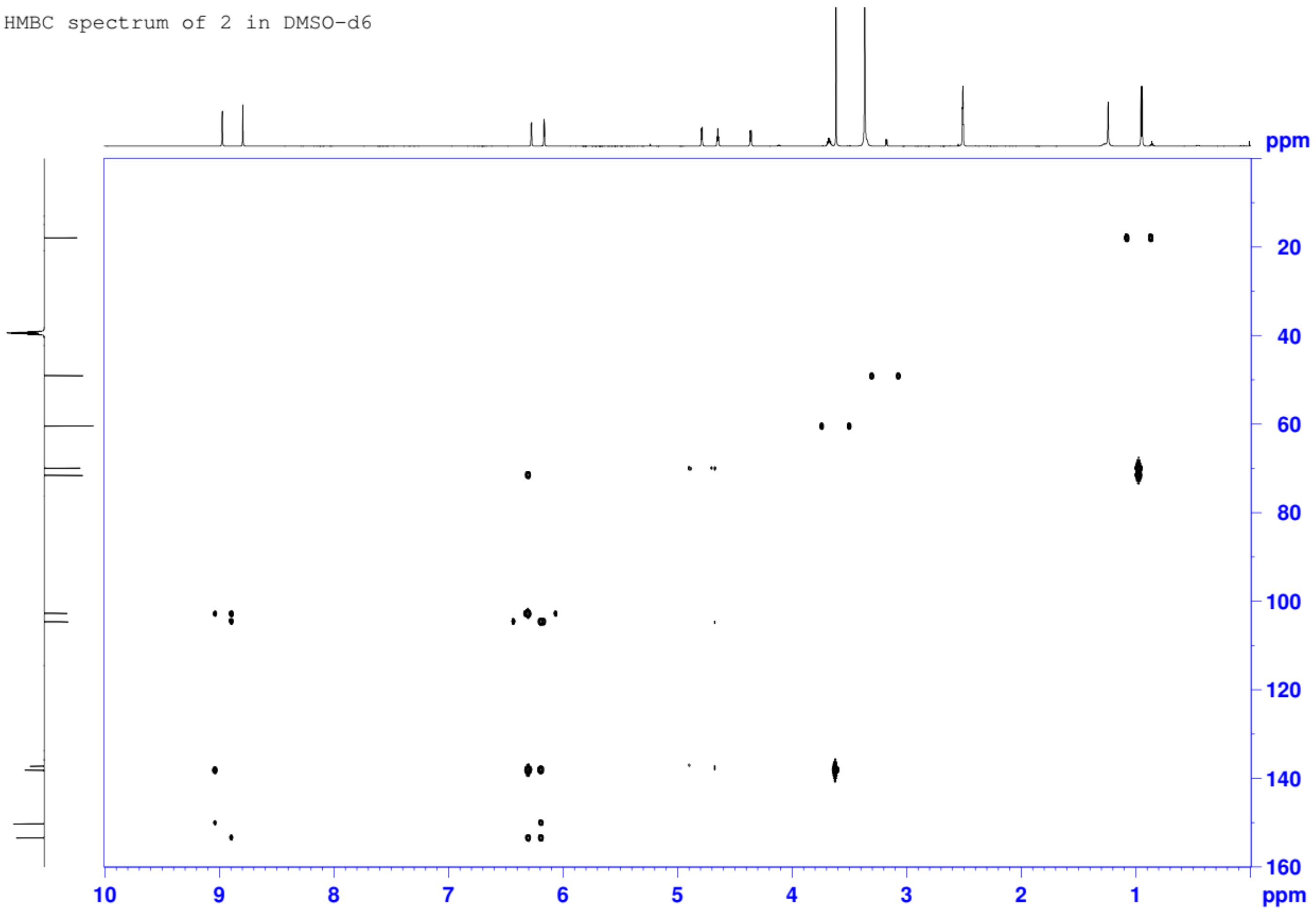

Figure S15. HMBC spectrum of **2** in DMSO-d_6.

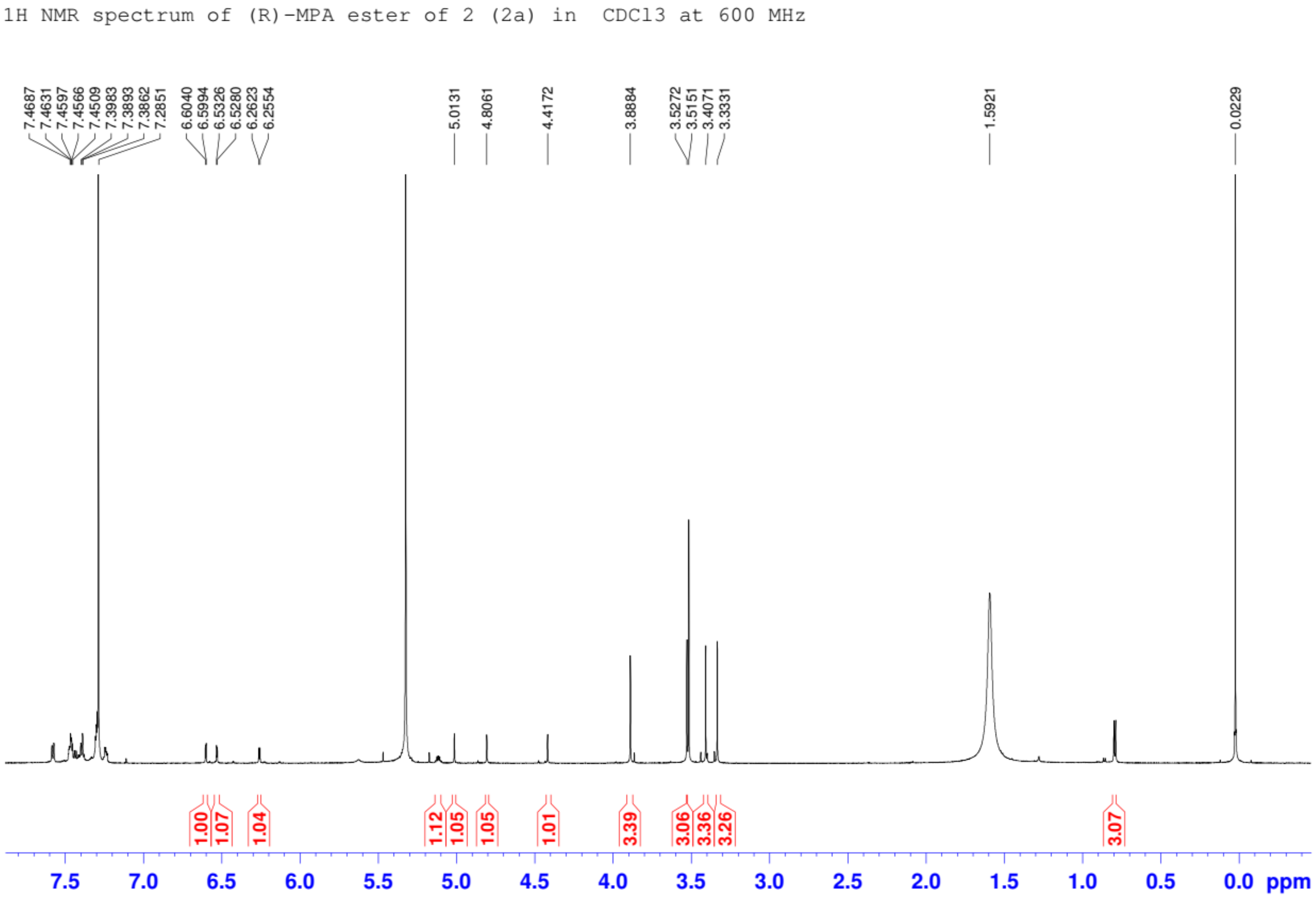

Figure S16. ^{1}H NMR spectrum of (*R*)-MPA ester of **2** (**2a**) in $CDCl_3$ at 600 MHz.

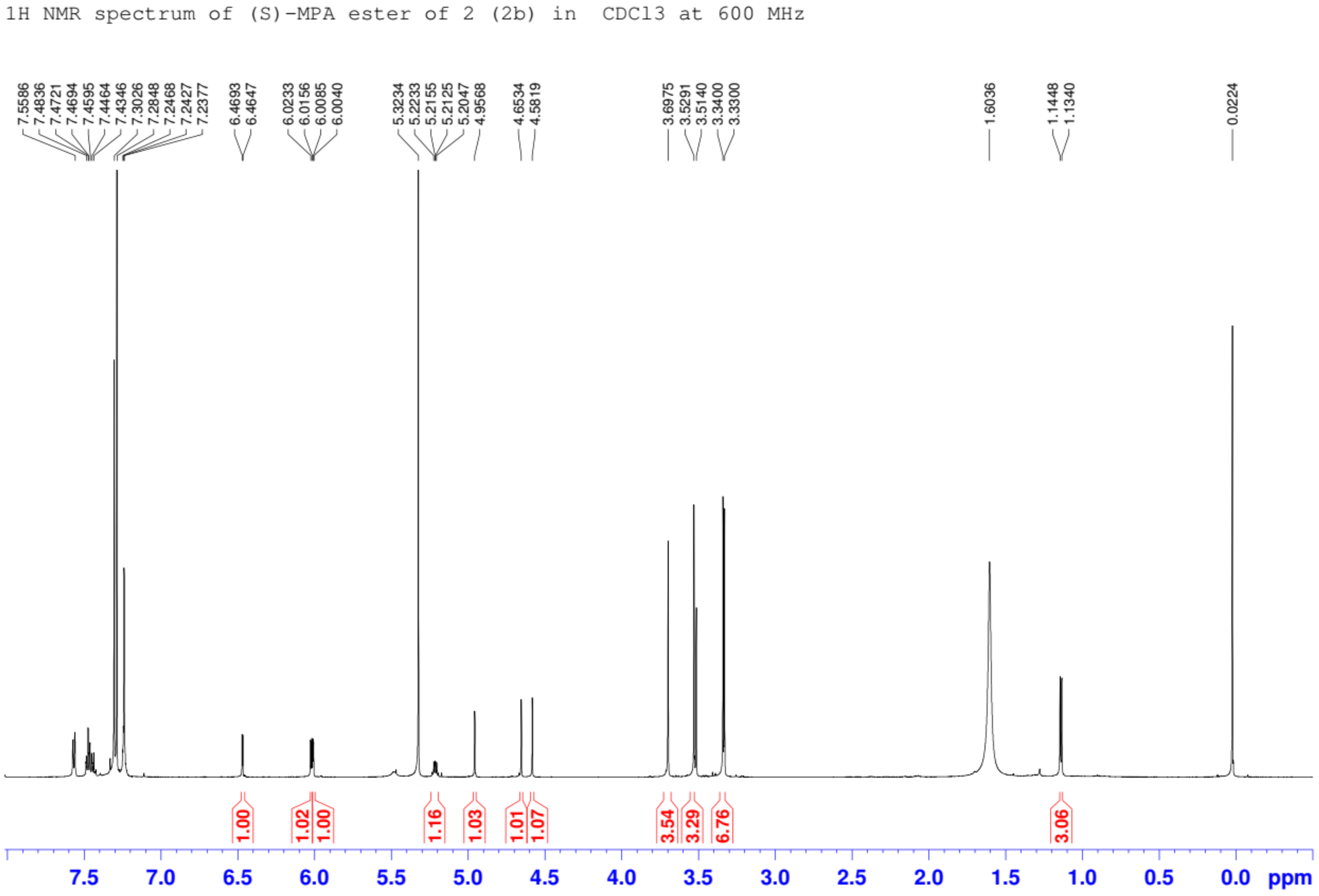

Figure S17. ^{1}H NMR spectrum of (*S*)-MPA ester of **2** (**2a**) in $CDCl_3$ at 600 MHz.

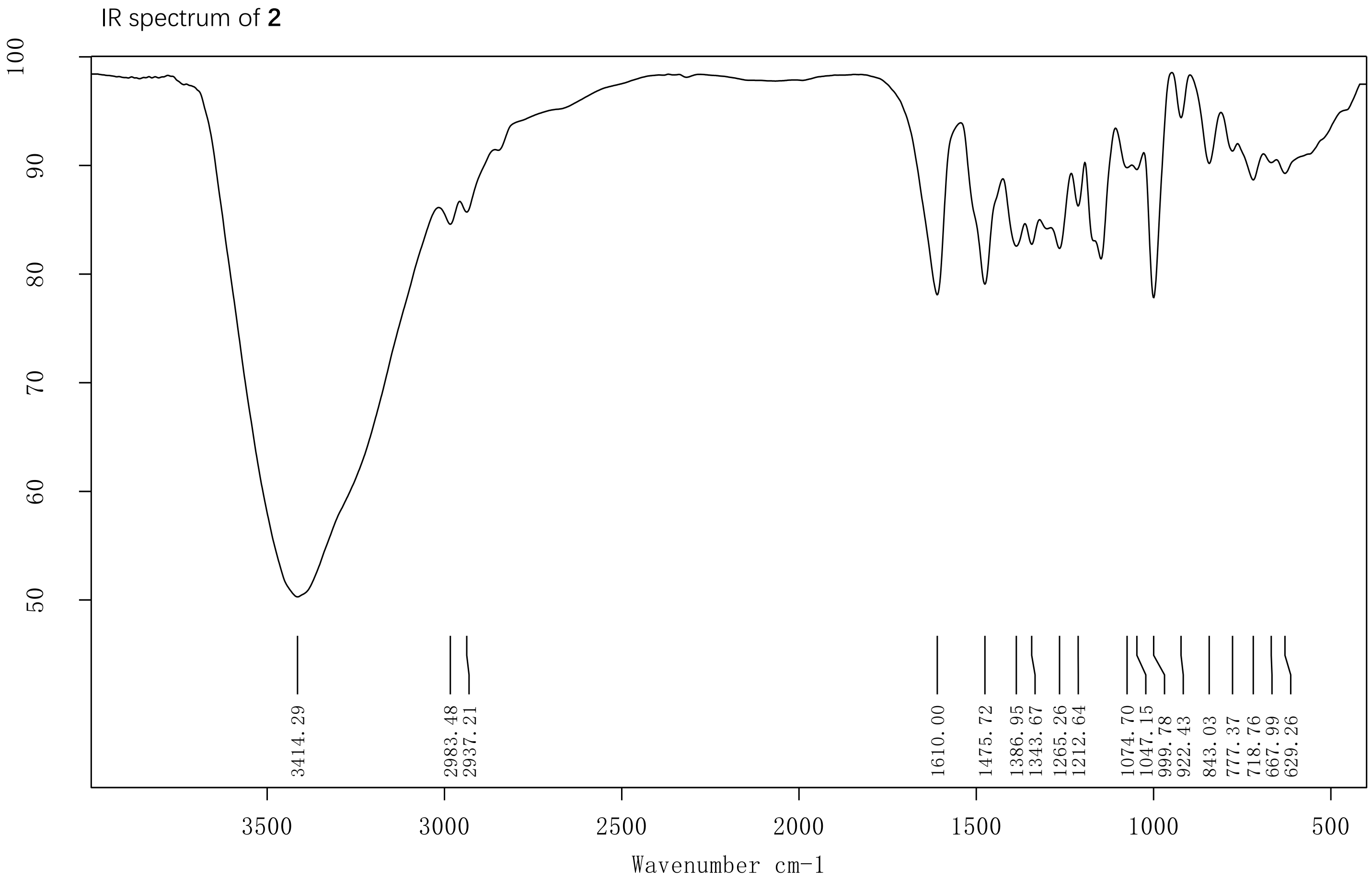

Figure S18. IR spectrum of **2**

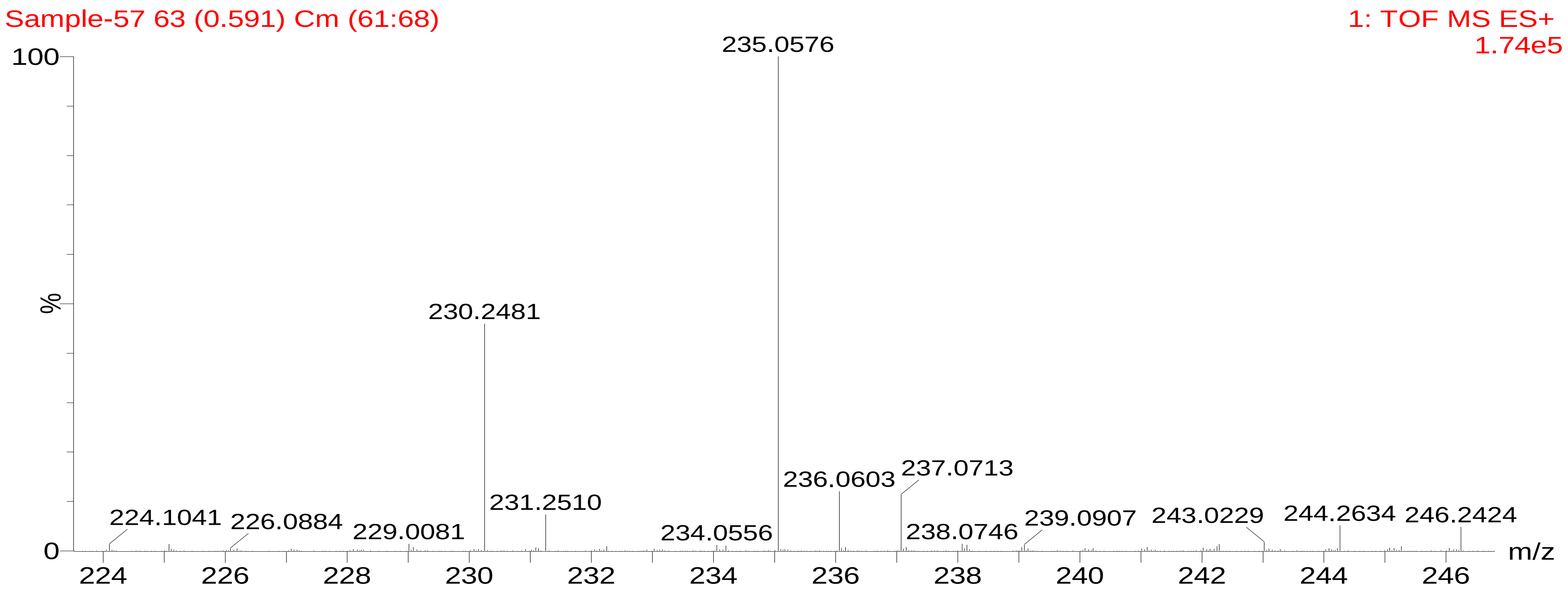

Figure S19. HRESIMS spectrum of **3**.

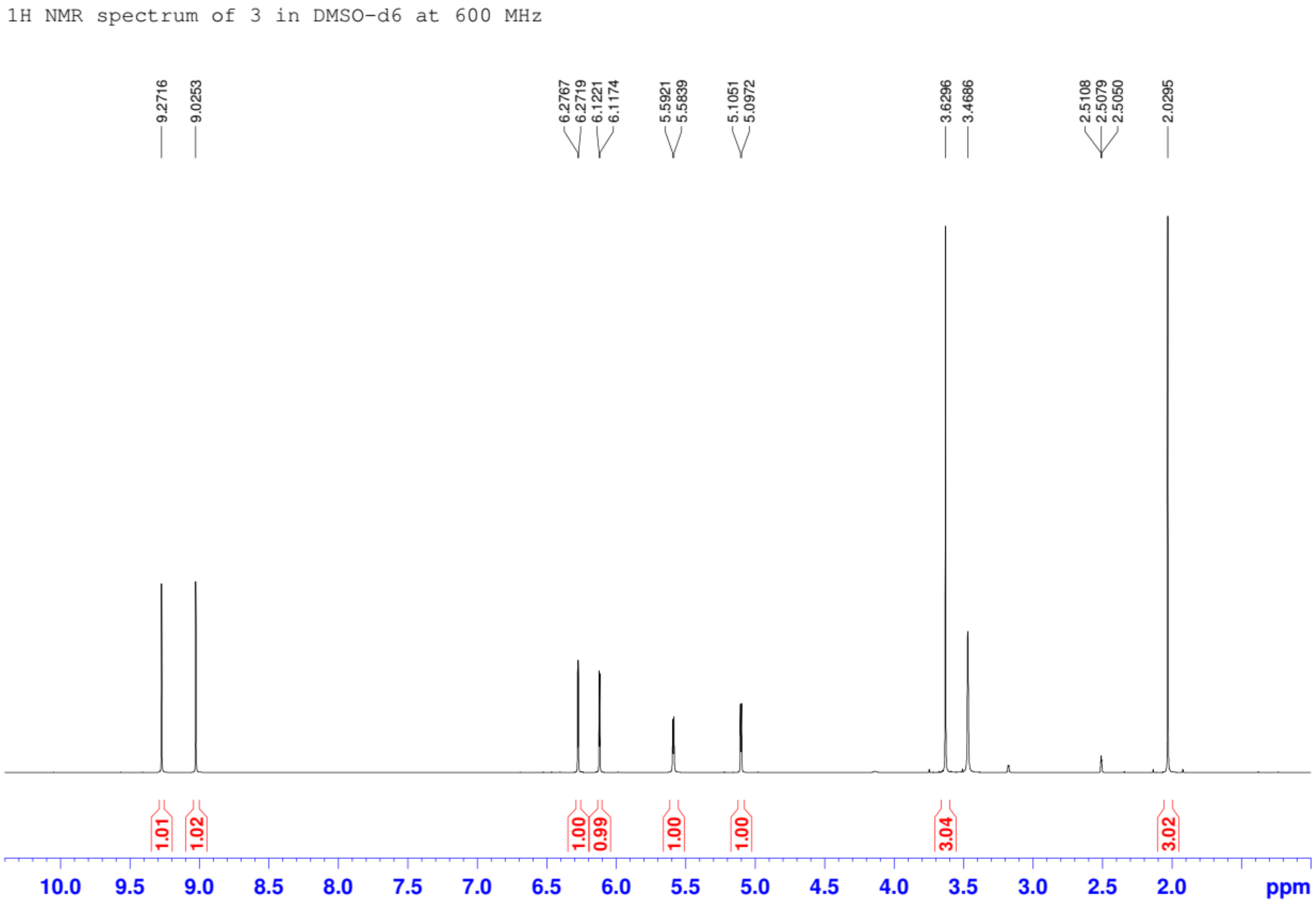

Figure S20. ^{1}H NMR spectrum of **3** in DMSO-d_6 (600 MHz).

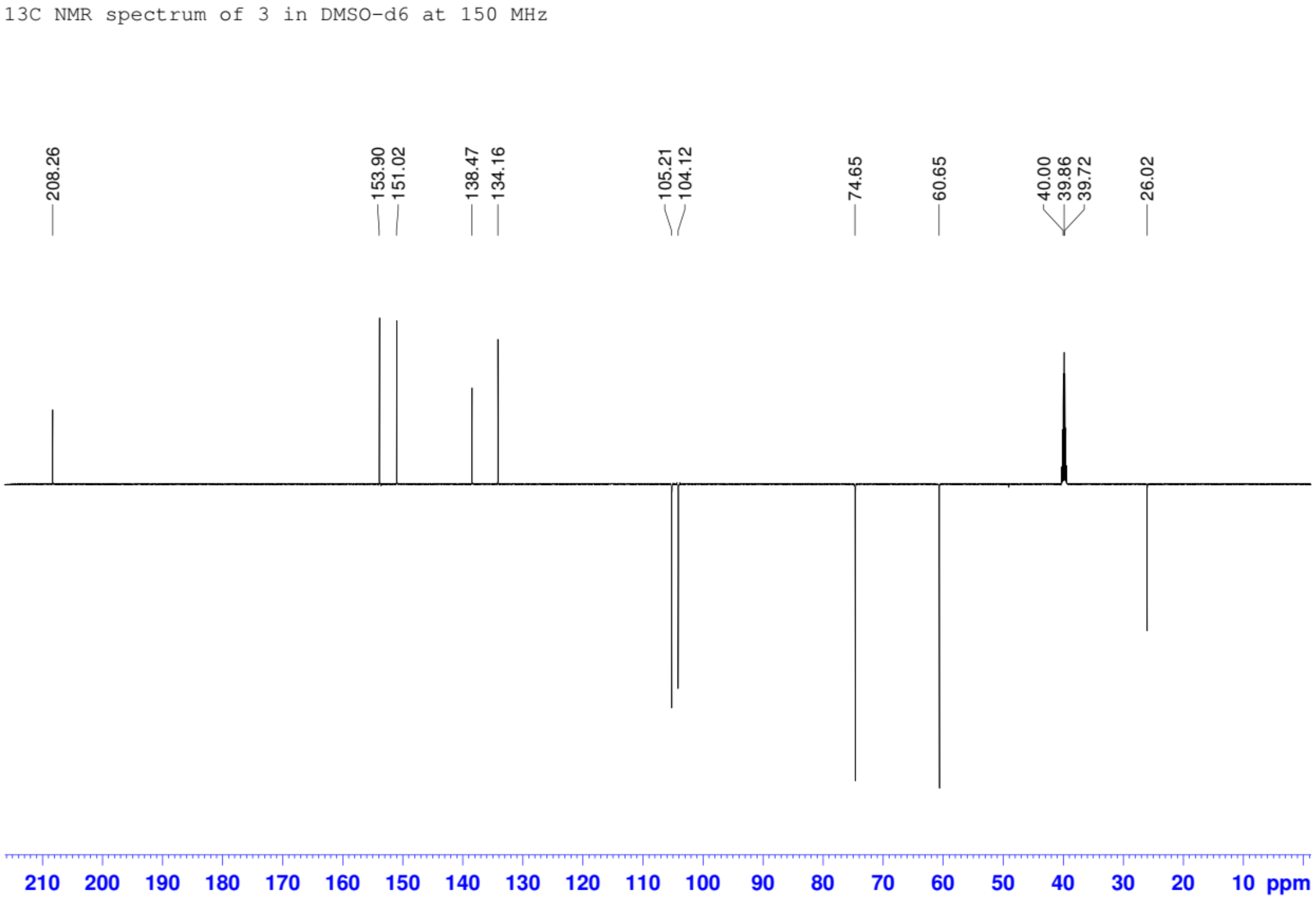

Figure S21. ^{13}C NMR spectrum of **3** in DMSO-d_6 (150 MHz).

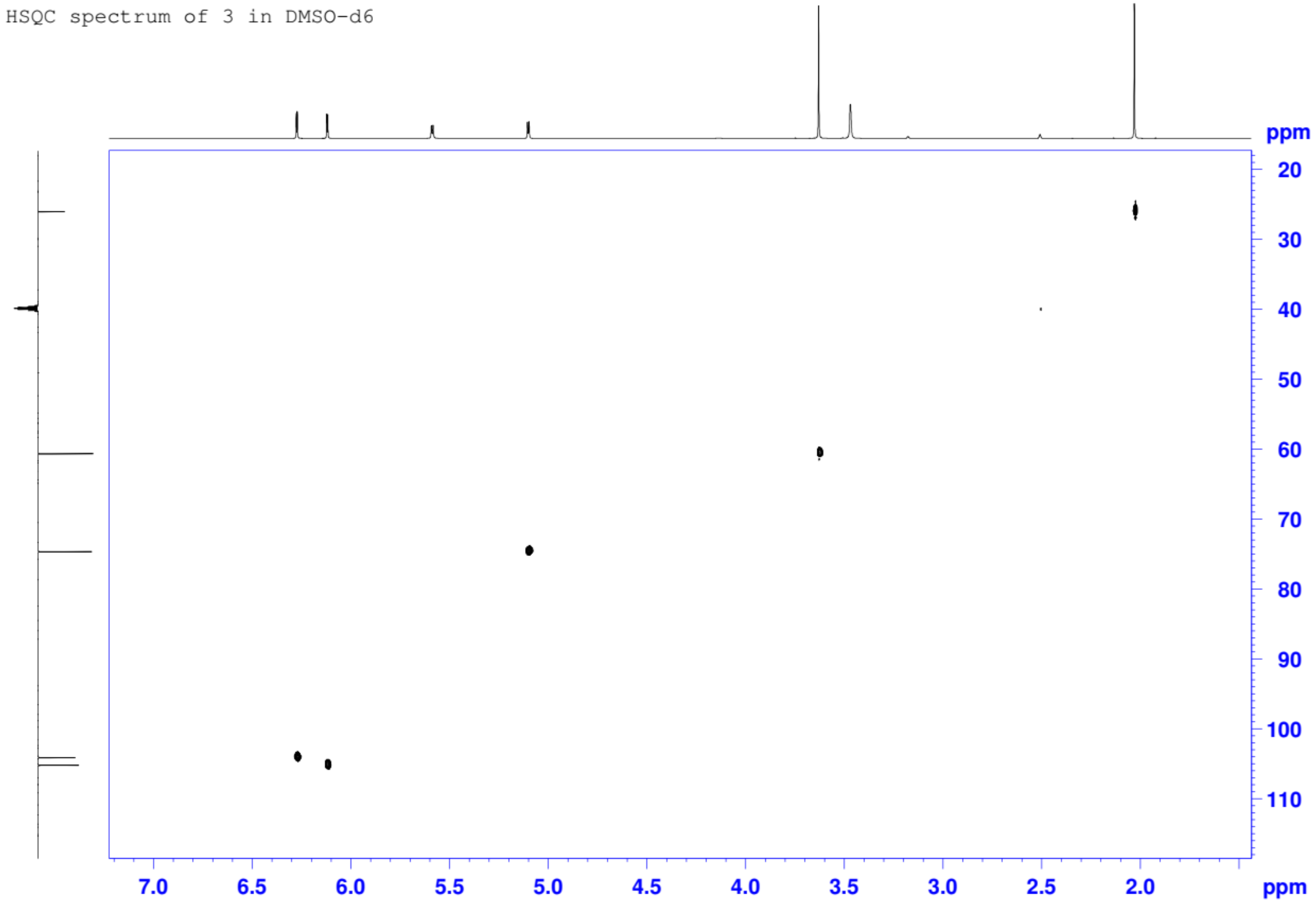

Figure S22. HSQC spectrum of **3** in DMSO-d_6.

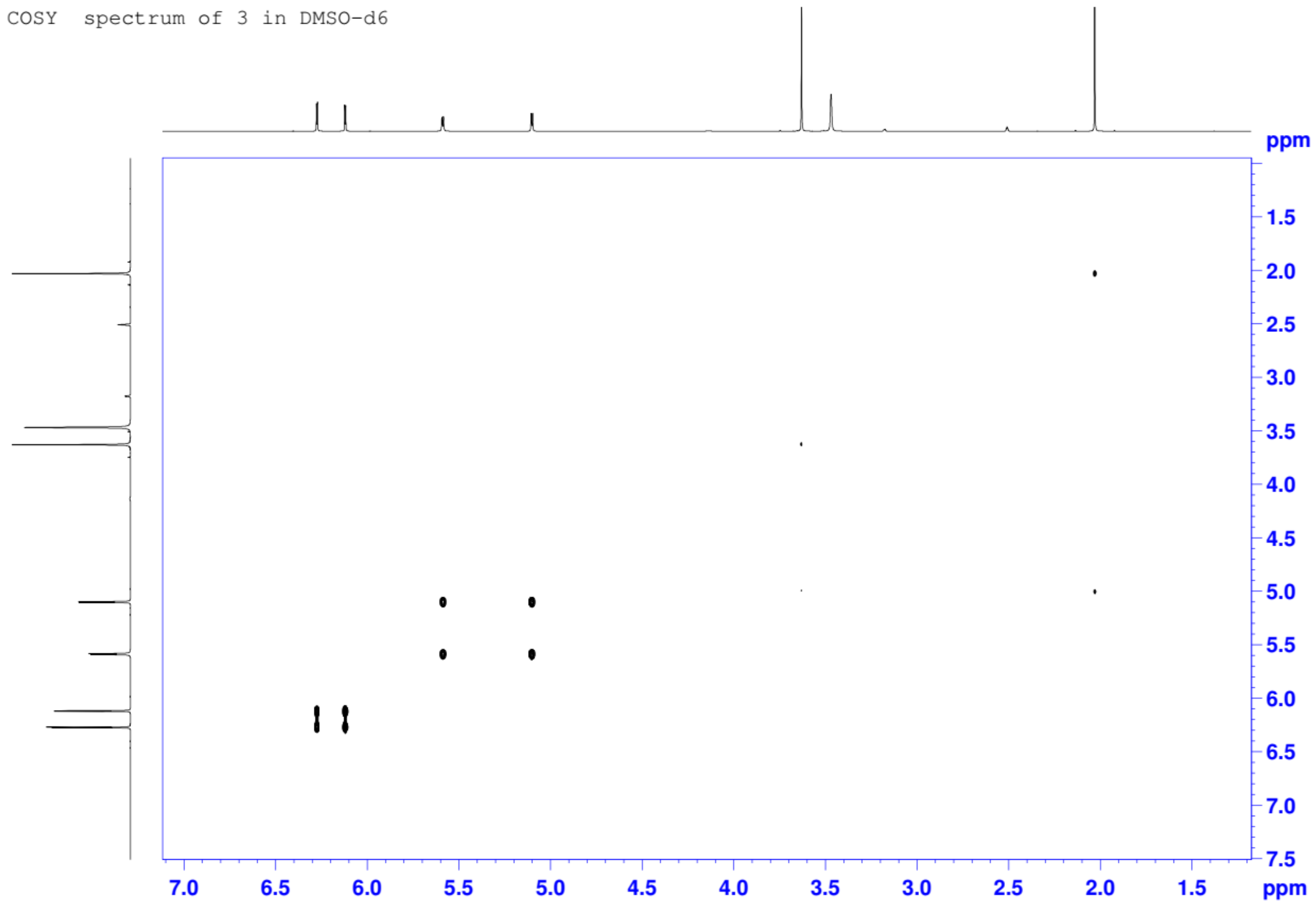

Figure S23. COSY spectrum of **3** in DMSO-*d*$_6$.

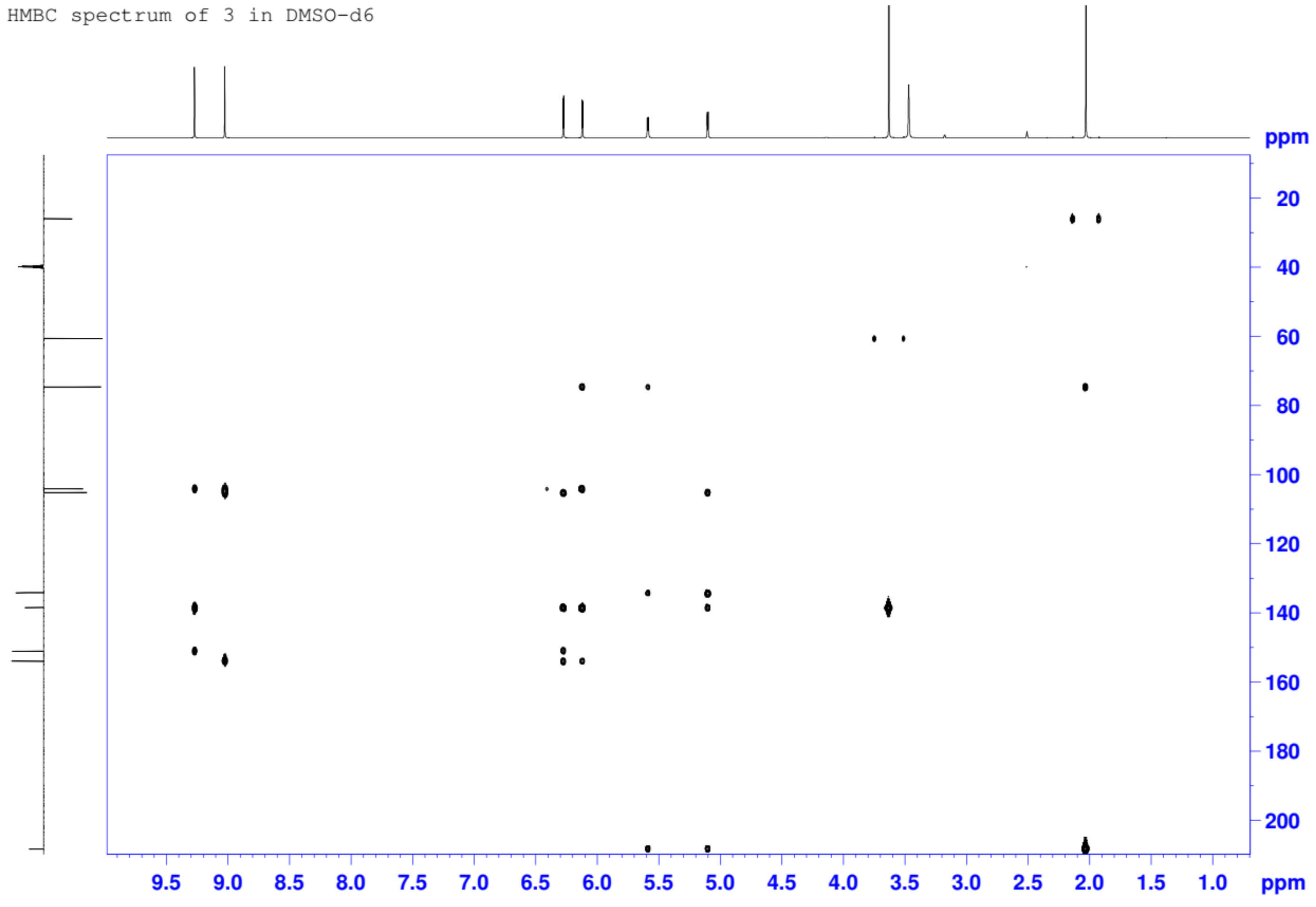

Figure S24. HMBC spectrum of **3** in DMSO-d_6.

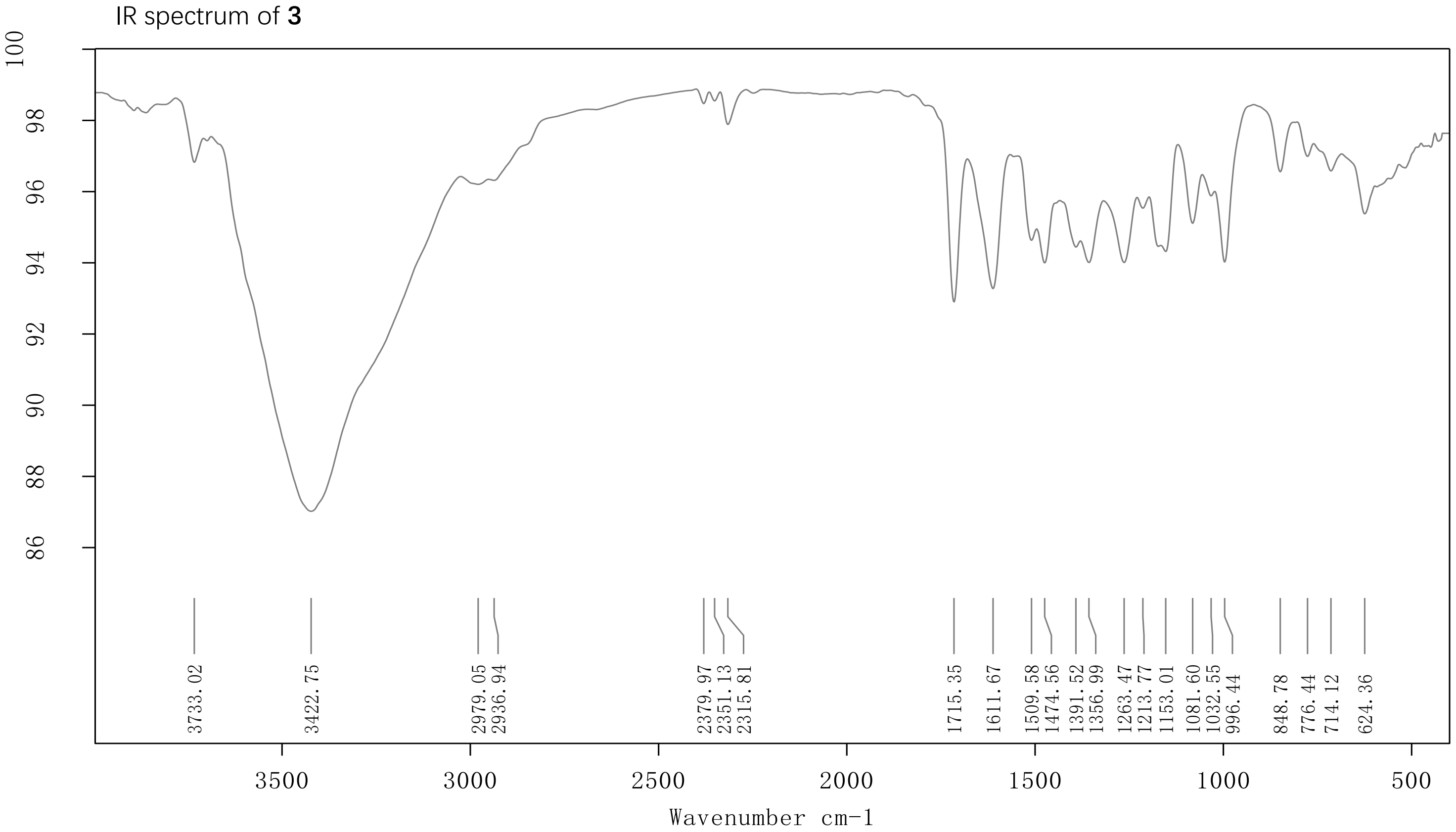

Figure S25. IR spectrum of **3**.

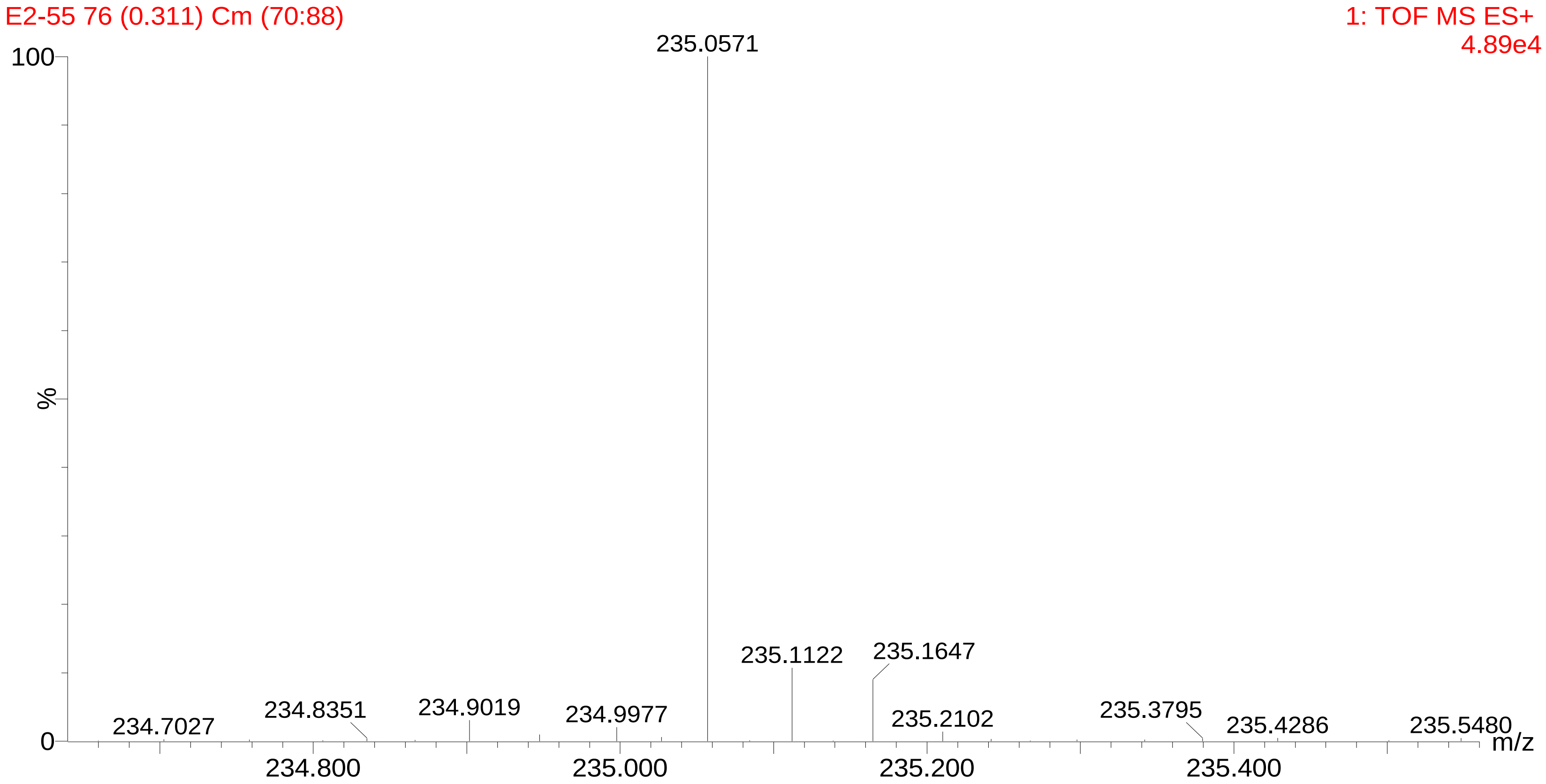

Figure S26. HRESIMS spectrum of **4**.

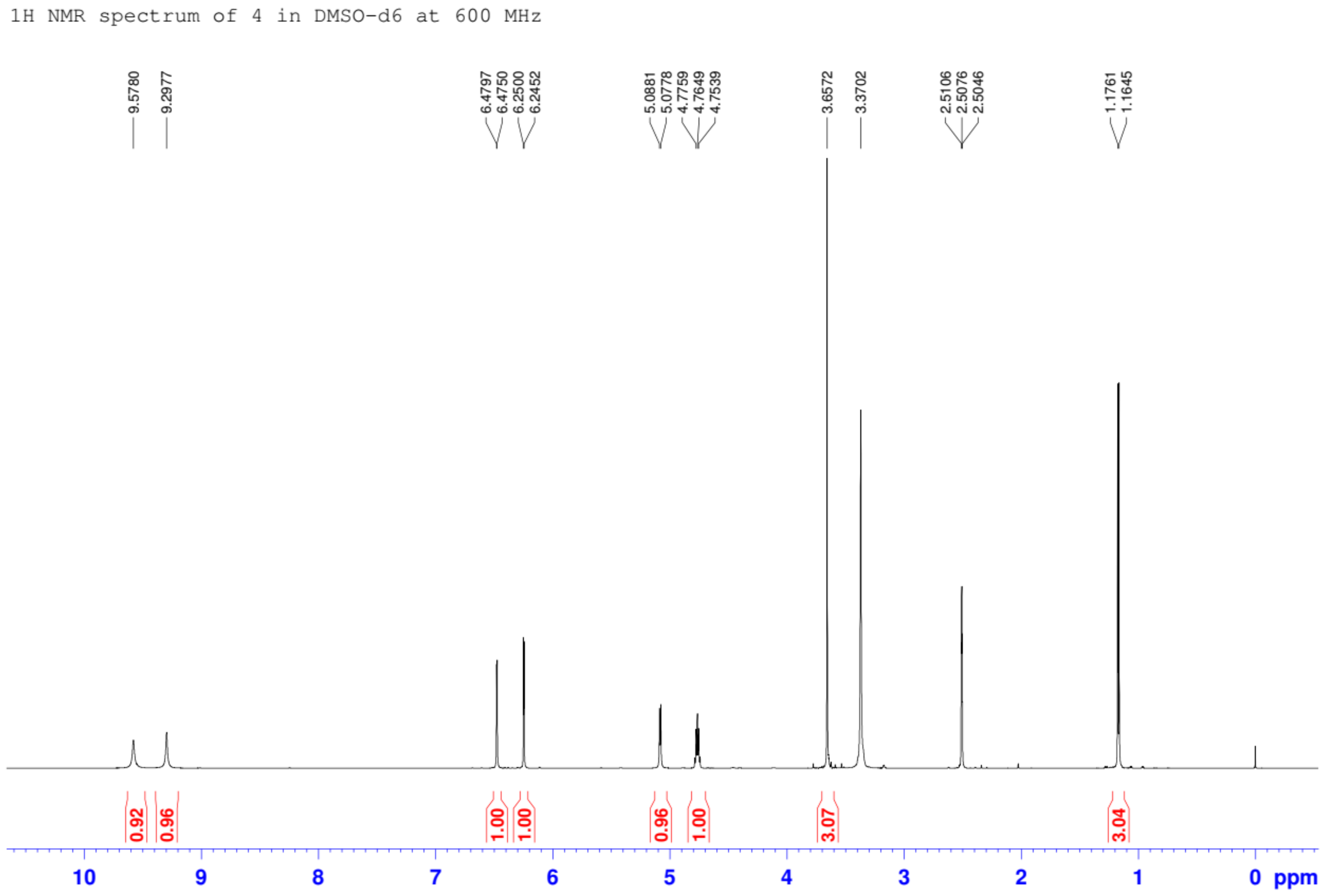

Figure S27. ^{1}H NMR spectrum of **4** in DMSO-d_6 (600 MHz).

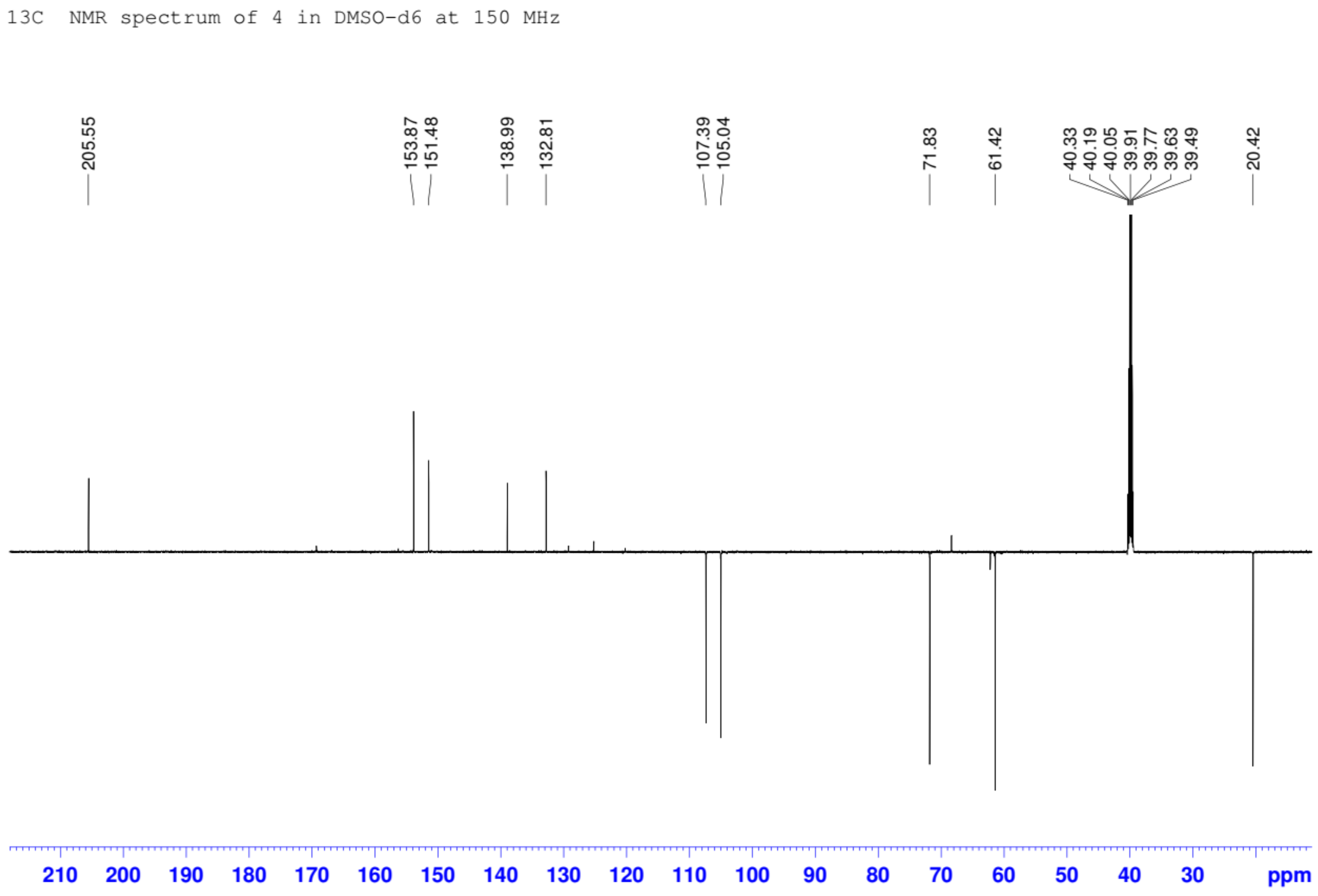

Figure S28. ^{13}C NMR spectrum of **4** in DMSO-d_6 (150 MHz).

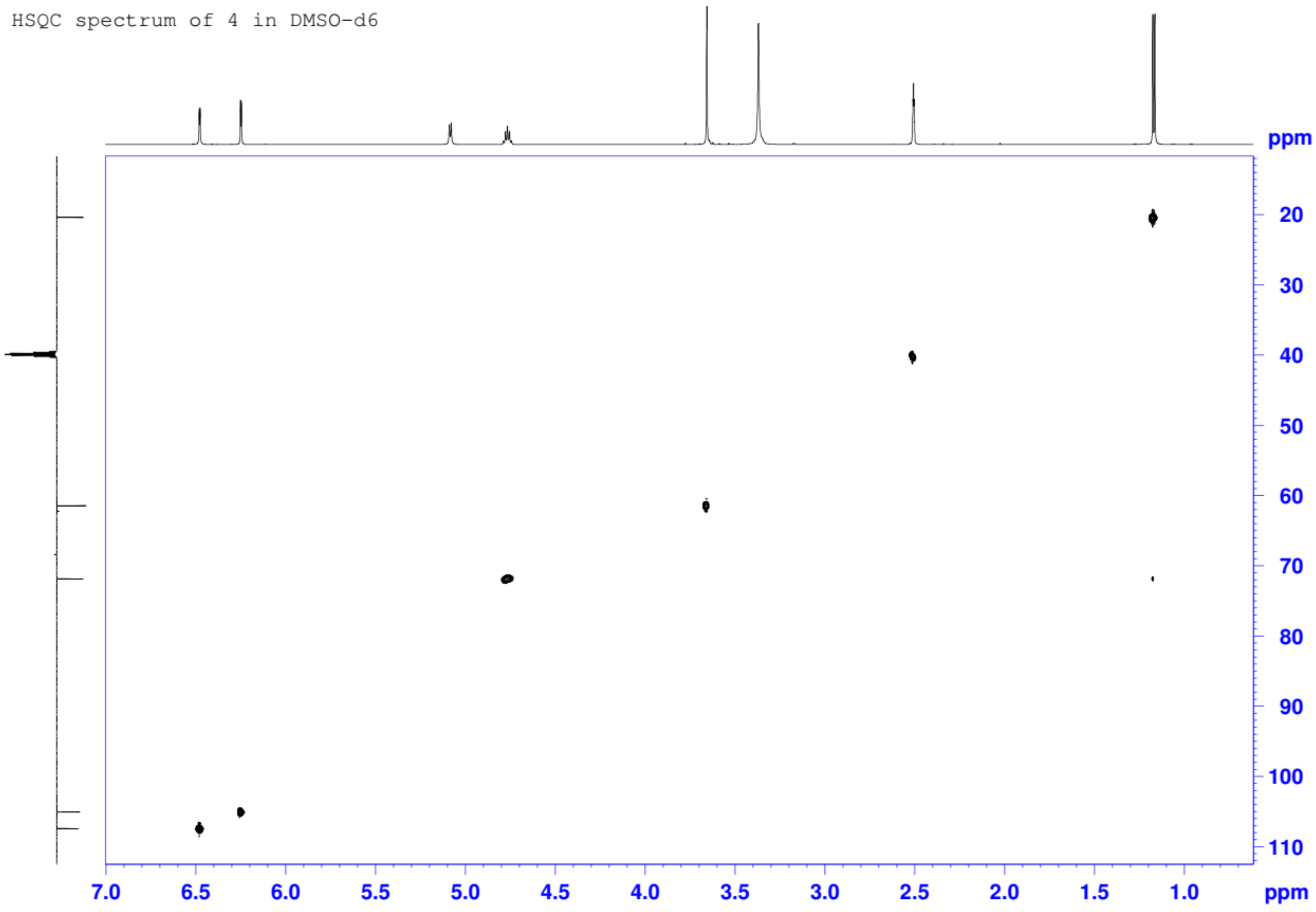

Figure S29. HSQC spectrum of **4** in DMSO-d_6.

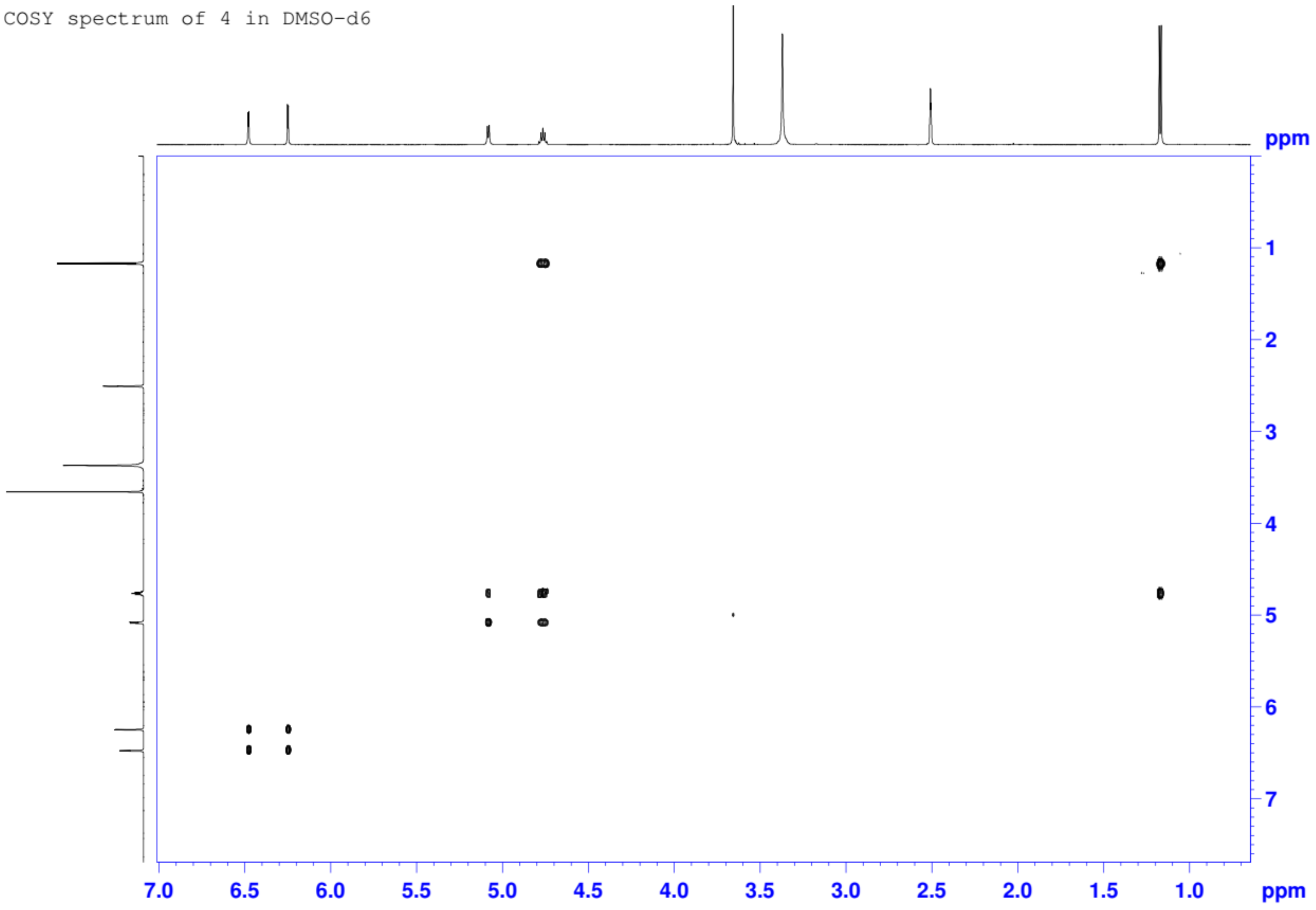

Figure S30. COSY spectrum of **4** in DMSO-d_6.

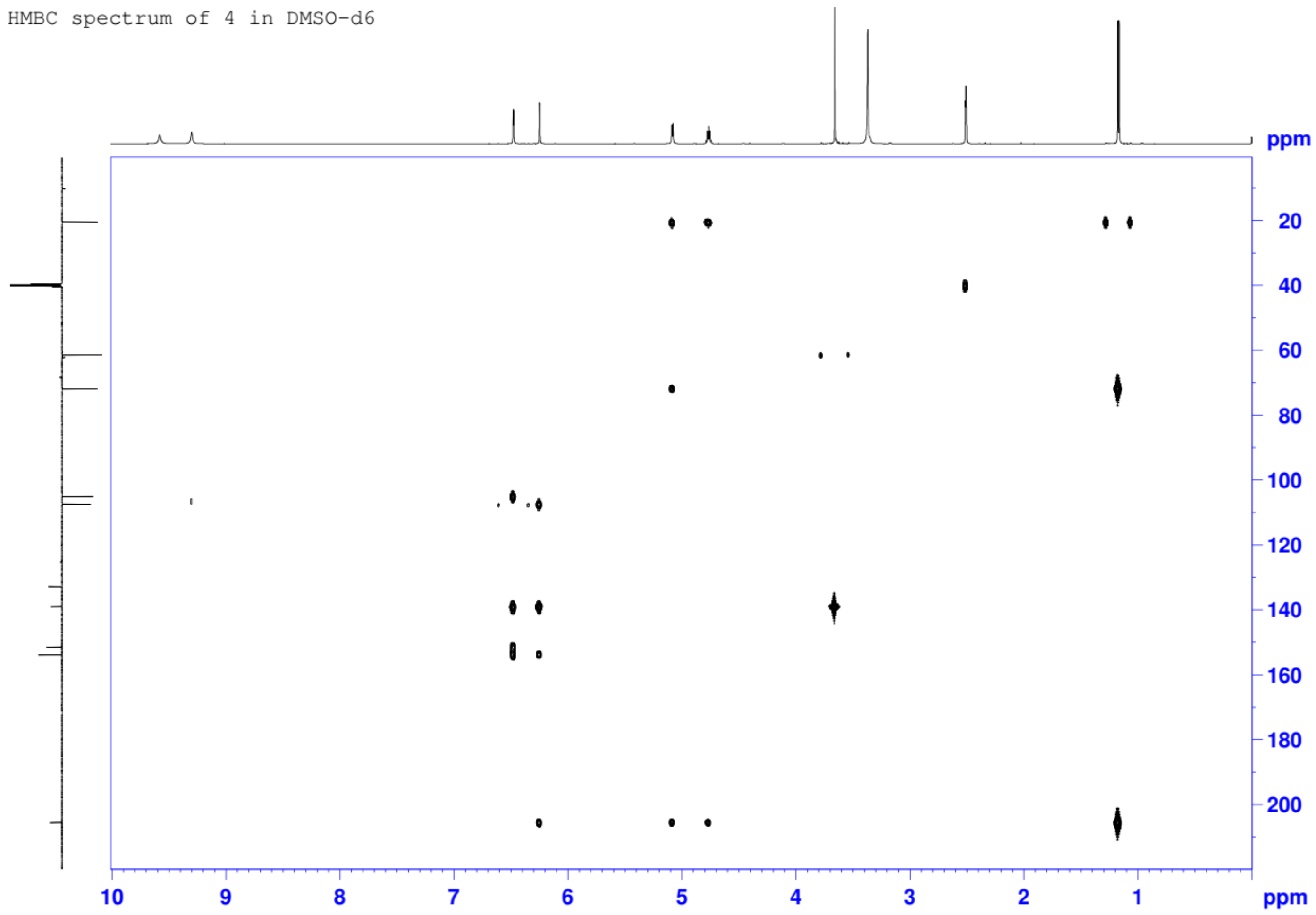

Figure S31. HMBC spectrum of **4** in DMSO-d_6.

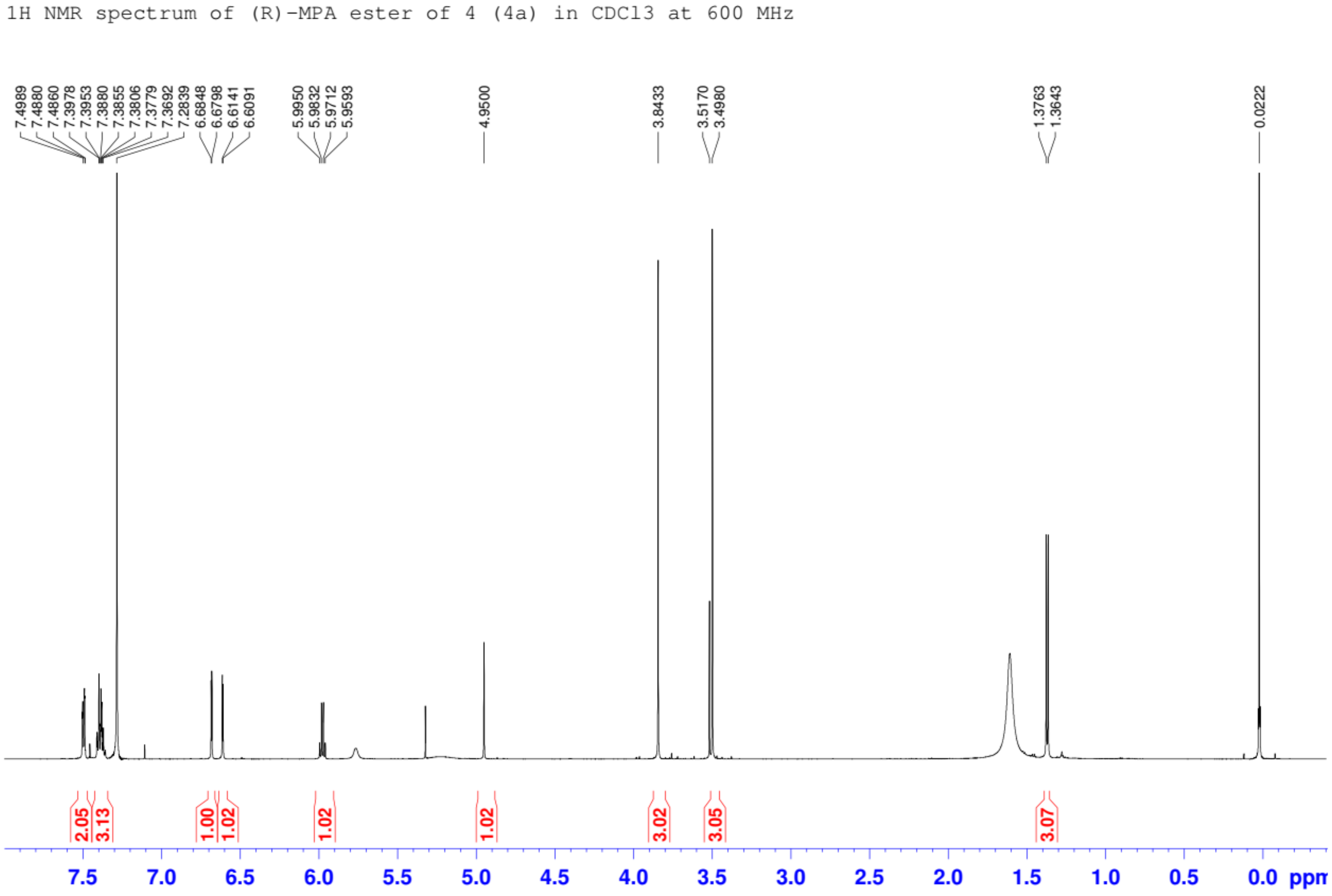

Figure S32. ^{1}H NMR spectrum of (*R*)-MPA ester of **4** (**4a**) in $CDCl_3$ at 600 MHz.

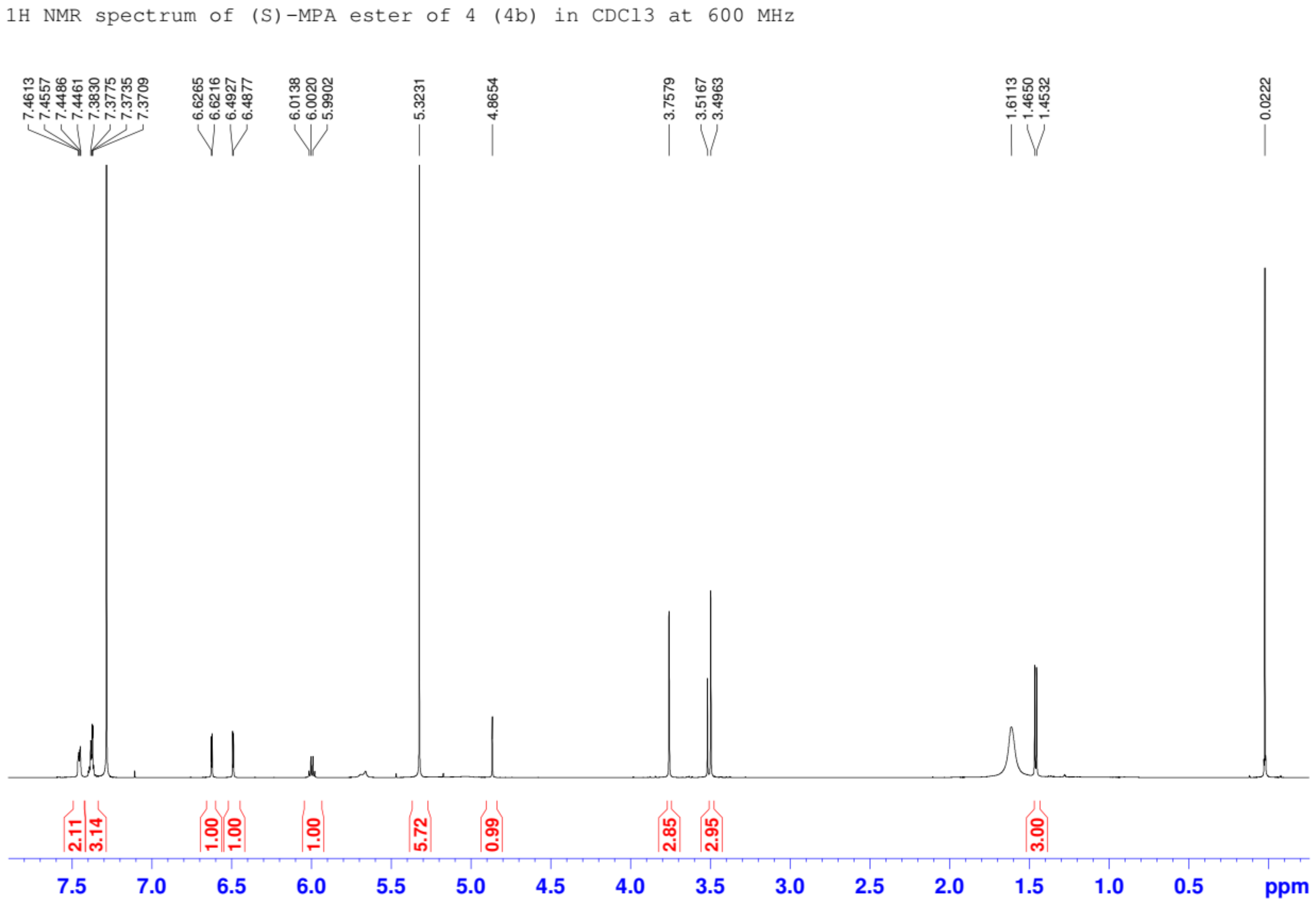

Figure S33. ^{1}H NMR spectrum of (*S*)-MPA ester of **4** (**4a**) in $CDCl_3$ at 600 MHz.

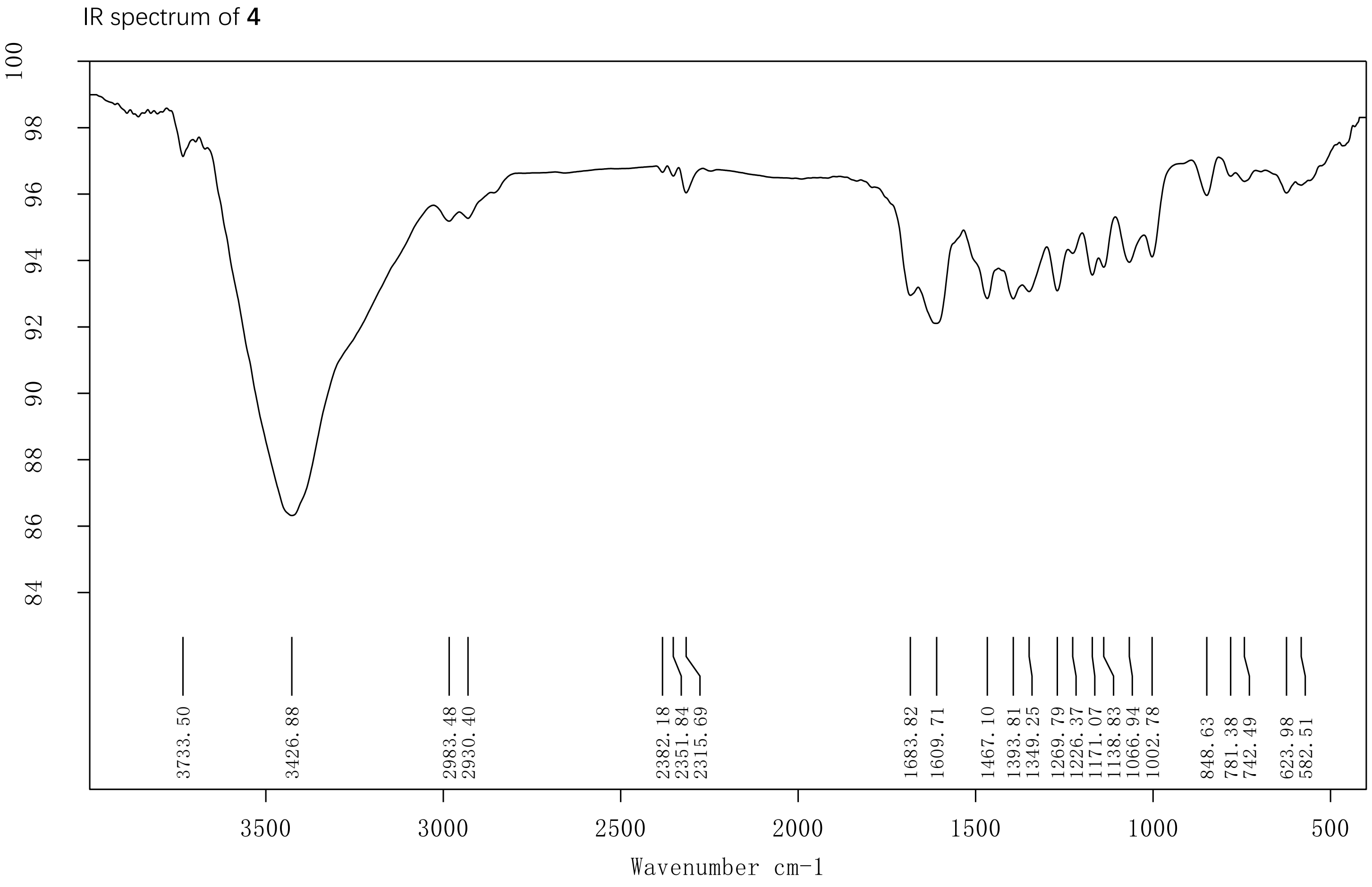

Figure S34. IR spectrum of **4**.
